# A chromosome-level genome assembly of the pollinating fig wasp *Valisia javana*

**DOI:** 10.1093/dnares/dsac014

**Published:** 2022-05-20

**Authors:** Lianfu Chen, Chao Feng, Rong Wang, Xiaojue Nong, Xiaoxia Deng, Xiaoyong Chen, Hui Yu

**Affiliations:** 1 Key Laboratory of Plant Resource Conservation and Sustainable Utilization, South China Botanical Garden, The Chinese Academy of Sciences, Guangzhou 510650, China; 2 Guangdong Provincial Key Laboratory of Applied Botany, South China Botanical Garden, The Chinese Academy of Sciences, Guangzhou 510650, China; 3 School of Ecological and Environmental Sciences, Tiantong National Station for Forest Ecosystem Research, East China Normal University, Shanghai 200241, China

**Keywords:** comparative genomics, *de novo* genome assembly, fig wasp, mutualism

## Abstract

Fig wasp has always been thought the species-specific pollinator for their host fig (Moraceae, *Ficus*) and constitute a model system with its host to study co-evolution and co-speciation. The availability of a high-quality genome will help to further reveal the mechanisms underlying these characteristics. Here, we present a high-quality chromosome-level genome for *Valisa javana* developed by a combination of PacBio long-read and Illumina short-read. The assembled genome size is 296.34 Mb from 13 contigs with a contig N50 length of 26.76 kb. Comparative genomic analysis revealed expanded and positively selected genes related to biological features that aid fig wasps living in syconium of its highly specific host. Protein-coding genes associated with chemosensory, detoxification and venom genes were identified. Several differentially expressed genes in transcriptome data of *V. javana* between odor-stimulated samples and the controls have been identified in some olfactory signal transduction pathways, e.g. olfactory transduction, cAMP, cGMP-PKG, Calcim, Ras and Rap1. This study provides a valuable genomic resource for a fig wasp, and sheds insight into further revealing the mechanisms underlying their adaptive traits to their hosts in different places and co-speciation with their host.

## 1. Introduction

Mutualism is ubiquitous in nature and at the core of the ecosystem, which has a profound impact on evolution at all levels of the ecosystem.^1–3^ Recently, specific mutualism, such as fig and fig wasp, yucca and yucca moth, has attracted extensive interest of researchers due to their high degree of interspecific adaptation and coevolution.^4^^,^^5^ The fig (Moraceae; *Ficus*) is pantropical and each species is likely pollinated by one to two wasps from the chalcid family Agaonidae. However, as many as nine pollinators can occur across a single host.^6^ The inflorescence of fig is an almost completely closed syconium, and has only one ostiole communicated with the outside. Only specialized fig wasps can enter it, lay eggs in the ovaries of the female flowers and pollinate some of female flowers at the same time. After some time, adults emerged and mated; then, the female fig wasps fly out of the syconium with pollen in search of new adaptive syconium to begin a new life cycle.^7^ Through a series of complex mechanisms, the mutual benefits between fig and fig wasps are maintained, and a high degree of synchronization and strict consistency are formed in morphology, behavior, physiology and development between them.^8^

Chemosensitivity mainly acts on the aspects of foraging, oviposition, mating, avoiding natural enemies and searching for hosts of insects.^9^ Fig wasps mainly rely on the accurate identification of volatile odors (VOCs) released from receptive syconium of their obligate host to find host.^10^^,^^11^ Each *Ficus* species produces a specific volatile blend that is only attractive to its specific pollinators.^12^^,^^13^ In addition to olfaction, other sensing, such as touch, taste and vision can also judge whether the host is suitable or not,^14^ which is mainly involved in odorant binding protein (OBP), chemosensory protein (CSP), olfactory receptor (OR), ionic receptor (IR) and gustatory receptor (GR).

The formation of olfactory sensation involves a series of complex signal transduction pathways. OBP transports VOCs to OR, and then OR activates.^9^ For insects, OR possess a distinct seven-transmembrane topology with the amino terminus located intracellularly and lack homology to G-protein-coupled chemosensory receptors in vertebrates.^15^,^16^ The activates of insects OR is either by the complex between it and its co-receptor (Orco) confers channel activity^16^ or by G-protein-mediated activated second messenger systems, such as cAMP and cGMP.^17^

Adult females of *Nasoniavitripennis* and other parasitic wasps in Chalcidoidea inject a venomous mixture into its host flies prior to oviposition to regulate host physiological processes, including immunity, developmental and metabolism, in order to protect and ensure offspring survival and successful development in the host.^18–20^ Although fig wasps are pollinators, they are actually parasitic in female flower ovary of the host. The developments of them are synchronous with that of host inflorescence.^21^^,^^22^ It has been reported that fig wasp inject venom into the ovary of female flowers to stimulate the production of endosperm to provide nutrition for the development of their eggs and larvae, and now the ovary is called gall.^23^^,^^24^ A gall is a proliferation of tissue abnormal for a site and generated by physiological manipulation of the plant through the insect.^8^ However, the venom gene of fig wasp has not been studied.

Highly species-specific lifestyle, behavior and living habit can be reflected at the genomic level.^25–27^ Given it has a specific symbiotic relationship with the host for tens of millions of years, fig wasp is now emerging as a model system for comparative genomics to study co-evolution and co-speciation.^28–30^ Three pollinating fig wasps, *Cerotosolen solmi,*^28^*Eupristina verticillate*^29^ and *Wiebesia pumilae*,^11^ have been sequenced at the genome level and annotated a large number genes about morphology, behavior, physiological and gender related, which were adapted to the highly specific symbiosis of fig wasps, and some gene families related to chemosensory, detoxification, innated immune response are reduced. There are more than 750 species of *Ficus* in the world.^31^ Although the species identification of fig wasps is relatively slow, there are more than 1000 species conservatively estimated (J. Y. Rasplus private communication). Therefore, more genetic studies on the genome level of fig wasp species can fully and deeply understand the interaction and evolution mechanism of fig wasp.

*Valisa javana* is an obligate pollinator of a dioecious fig, *Ficus hirta*, which is widely distributed in Southeast Asia. Due to its typical asynchronous flowering phenology and located under the canopy, *V. javana* tends to pollinate within the population or even within a tree of its host, so its flight distance should be relatively limited and more likely to speciation by geographical isolation. The species complex occurred in *F. hirta* across Southeast Asia and eight of them are related.^6^ In order to better understand the mechanism of speciation and differentiation of these related species, we need to have a general understanding of their genomics.

Here, we report a high-quality chromosome-level genome assembly for one of species of the complex, *V. javana* complex sp. 1, using a combination of PacBio long-read sequencing and Illumina short-read sequencing. The assembly has high completeness, providing an excellent genomic resource for subsequent research. In this study, besides the basic genome description and comparative genomics, we identified chemosensory genes, detoxification, venom genes and several differentially expressed genes (DEGs) in *V. javana* related to olfactory sensing. This genome assembly serves as a useful resource for further research into insect biology, pollinator–host interactions, comparative genomics and coevolution.

## 2. Materials and methods

### 2.1. Genome sequencing

For a receptive male syconium of *F. hirta*, only one or a few female fig wasps can enter and oviposit there.^32^ So, the pollinating wasps from one syconium are mainly the offspring of one or a few mothers. We have once checked the pollinator species of *F. hirta* in Guangdong Province by DNA sequencing and morphology based on plenty of samples,^6^^,^^33^^,^^34^ and they are all *V. javana* complex sp. 1.^6^

Nearly, 500–1,000 female adult individuals of *V. javana* were collected from several figs of *F. hirta* in a single tree in Baiyun Mountain, Guangdong Province of China for DNA extraction, libraries construction and whole-genome sequencing. DNA was extracted with Easy Pure Genomic DNA Extraction Kit (Beijing, China).^33^

The paired-end libraries with insert size of ∼310 bp were constructed using VAHTS Universal DNA Library Prep Kit and then sequenced on an Illumina Hiseq4000 platform (San Diego, CA, USA). The raw Illumina reads were purified with Trimmomatic 0.39^35^ and then used for downstream genome alignment. In addition, more than 10–15 μg of sheared and concentrated DNA was applied to size-selection by BluePippin system. Long DNA fragments of ∼20 kb SMRTbell™ libraries were constructed with Sequel Sequencing Kit 2.0. A total of four single-molecule real-time cells were sequenced on Pacbio RSII system.

### 2.2. Genome assembly

Canu,^36^ FALCON,^37^ DBG2OLC^38^ and NextDenovo (https://github.com/Nextomics/NextDenovo, 22 May 2022, date last accessed) were used for contig assembling. At last, N50 size of 26.74 Mb was got from NextDenovo for next genome optimization. After 18 duplicated contigs and 6 haplotype contigs were filtered by Purge_dups version 1.2.5,^39^ the retained 26 contigs were polished with full PacBio subreads and Illumina clean reads by NextPolish version 1.3.1 according to default parameter.^40^

### 2.3. Assessment of the genome completeness and quality

The completeness of the genome was evaluated through estimating the genome size, BUSCO (Benchmarking Universal Single-Copy Orthologues) analysis and genome coverage calculation.

The clean data of Illumina reads were used for *K*-mer distribution analysis by GenomeScope2.0 version 1.0.0^41^ for genome survey. With *K*-mer size set to 21 and ploidy set to haplotype, the genome size was estimated to be 304 Mb.

BUSCO version 4.1.2^42^ was used to evaluate the completeness of the contigs assembly and whole-genome proteins separately based on the hymenoptera_odb10 database which including 5,991 BUSCOs. Besides, we also performed BUSCO analysis using whole-genome proteins of 29 other insect species based on corresponding databases (endopterygota_odb10 for Coleoptera species, diptera_odb10 for Diptera, hymenoptera_odb10 for Hymenoptera and hemiptera_odb10 for Hemiptera).

The genome coverage was calculated by mapping the Illumina reads to a reference genome using Bowtie2 version 2.3.5.^43^

### 2.4. Repeat annotation

We detected repetitive sequences and transposable elements in the genome using a combination of *de novo* and homology-based approaches. Repetitive sequences appeared at least 16 times were searched by *ab inito* algorithm using RepeatModeler version 2.0.1.^44^ The conserved transposons were searched by RepeatMasker version 4.1.0^45^ according to the Insecta repeats within RepBase database.^46^

### 2.5. Non-coding genes annotation

The non-coding genes including tRNAs, rRNAs, snRNAs and miRNAs were mainly annotated by aligning the genomic sequence against the covariance models (CMs) of RFAM database version 14.5^47^ with Infernal version 1.1.2.^48^ In contrast to only one CM of tRNA in Rfam database, more refined eukaryotic CMs were used by tRNAScan-SE version 2.0.2^49^ for a more accurate prediction.

### 2.6. Gene prediction and functional annotation

Protein-coding genes were predicted by combing RNA sequencing (RNA-seq)-based, homolog-based and *ab initio* methods performed on the repeat masked genome. For the RNA-seq-based method, short reads from transcriptome sequencing were aligned to the genome with Hisat2 version 2.1.0^50^ and the gene structures were built by Sam2transfrag, a module of GETA version 2.4.6 (https://github.com/chenlianfu/geta, 22 May 2022, date last accessed). The homologous proteins from the whole genome of three fig wasp species, *W.pumila*, *Ceratosolen solmsi*, *Eupristina verticillata*, jewel wasp *N.vitripennis*, *Copidosoma floridanum* and fruit fly *Drosophila melanogaster* were employed to predict gene structures by genewise version 2.4.1.^51^ Then the derived complete or partial gene models were used to train Augustus HMM (Hidden-Markov Model) parameters and predicted genes *ab initio* assisted by the hints of intron, CDS, exon, start and stop codon using AUGUSTUS version 3.3.3.^52^ The results of three methods were integrated, and the gene models were filtered if it couldn’t match to a HMM model from eukaryotic HMM database of eggNOG version 5.0.^53^

The functional annotation of protein-coding genes was performed by aligning protein sequences to Nr,^54^ Swiss-Prot,^55^ KOG^56^ and eggNOG version 5.0^53^ using BLASTP method of Diamond 2.0.9.147.^57^ Furthermore, the databases of InterPro version 85.0^58^ and Pfam version 34.0^59^ were used for gene family and domain annotation by Interproscan 5.51^58^ and Hmmer 3.3.2^60^ separately. The genes annotated from eggNOG and InterPro were integrated by Gene Ontology (GO). The KEGG (Kyoto Encyclopedia of Genes ad Genomes) annotation were employed by web tool KAAS.^61^

### 2.7. RNA-Seq and analysis

In total, six RNA-Seq libraries were constructed, including three biological replicate samples of *V. javana* which were stimulated by odor emitted by B-phase male syconia of its host for at least 30 min and three biological replicates of controls. Approximate 50–60 female adult individuals for each sample were collected for RNA extraction, libraries construction and RNA-Seq. RNA was isolated using TRIzol™ (Tiangen). For each sample, a messenger RNA (mRNA)-Seq library was constructed using an Illumina TruSeq™ RNA Sample Preparation Kit (Illumina, San Diego, CA, USA) following the manufacturer’s recommendations. The isolation of mRNA, fragment interruption, complementary DNA (cDNA) synthesis, adaptor ligation, PCR amplification and RNA-Seq were performed by Novogene Bioinformatics Technology Co., Ltd. (Beijing, China). The transcriptomes were sequenced using the Illumina HiSeq 2000 platform with paired-end libraries.

Low-quality reads were filtered using Trimmomatic version 0.38.^35^ The RNA-seq clean reads were mapped to the *V. javana* genome using Hisat2 version 2.1.0.^50^ Then the alignments were processed for gene counts calculation by HTseq version 0.11.2.^62^ The raw counts of all samples were normalized across transfer matrix method (TMM) algorithm to get gene expressions.^63^ After low expression genes filtered with threshold, the false discovery rate (FDR) values of genes were calculated by EdgeR 3.30.3^64^ and DEseq2 1.28.1,^65^ and then DEGs were identified. The analysis of GO and KEGG pathway enrichment for DEGs were performed by Fisher’s exact test. One of stimulated samples was an outlier in almost all the pairwise comparisons and made the number of DEGs very low. Therefore, an additional analysis was done including only two stimulated samples.

### 2.8. Identification of ortholog and phylogenetic analysis

To find orthologous genes, genome the coding sequence (CDS) and protein sequences of 14 Hymenoptera species (including four fig wasp species, *V.javana*, *W.pumilae*, *E.verticillata* and *C.solmsi*), four Diptera species (including *D.melanogaster*), four Lepidoptera species, four Coleoptera species and four Hemiptera species were downloaded from NCBI (Supplementary Table S1). Pairwise comparison of protein sequences was made using BLASTP in Diamond 2.0.9.147^57^ and filtered by in-house perl script. Orthologous gene pairs were found using OrthoMCL 2.0.9^66^ and clustered by MCL algorithm.^67^ Each orthologous cluster group (OCG) represents a gene family and contains genes from at least two species. Functional annotations including Nr, Swiss-Prot, KOG, eggNOG, Pfam, KAAS and GO were performed, and the OCG obtained responding functional annotations when ≥30% of its genes had the same annotation.

We reconstructed a phylogeny for 30 insects using the single-copy genes from OrthoMCL results. The protein sequences of every species in each OCG were independently aligned by MAFFT version 7.407.^68^ These sequences were then transferred to CDS sequences using in-house perl script and retained the conserved blocks ≥60 bp using Gblocks 0.91 b.^69^ At last, the aligned conserved codon sequences of 1,467 single-copy genes were concatenated to obtain the maximum likelihood (ML) tree using RAxML version 8.2.12^70^ with bootstrap set to 300. The divergence time among species was calculated by MCMCTree in PAML version 4.9i.^71^ Three calibration time points from TimeTree database,^72^ stem Hemipter (177–401 MYa), stem Diptera (217–301 MYa) and stem Lepidoptera (80–157 MYa) were used for divergence time calibration. The tree was visualized using figtree version 1.4.4 (http://tree.bio.ed.ac.uk/software/figtree/, 22 May 2022, date last accessed).

### 2.9. Genome synteny

According to the topological structure of the phylogenetic tree, the evolutionary relationship between *V. javana* and *W. pumila* is the closest. So genome synteny between *V. javana* and *W. pumila* was detected by MCScanX^73^ with default parameters and plotted using Circos version 0.69-6.^74^

### 2.10. Genes under positive selection

In total, 16,098 OCGs present in at least 4 species were analyzed potentially experienced positive selection. First, codon alignment was transferred and an unrooted sub-tree was extracted from the phylogenetic tree for each OCG. Second, dN/dS was calculated for the pairwise comparison between all codon sequences of each OCG using in PAML version 4.9i^71^ and choose the OCGs with dN/dS > 1.0 as candidate genes. Combining the union set of the above two algorithms, 66 candidate OCGs are obtained as candidate positive selection genes for the following analysis: for these genes, the subtree information containing only the corresponding species was extracted according to the phylogenetic tree. Then the positive selection analysis was carried out together with the results of codon multi sequence alignment. The branch-site model A (model = 2 && NSsites = 2) and modified branch-site model A (null model, model = 2 && NSsites = 2 && fix_omega = 1 && omega = 1) were performed to selected the candidate genes under positive selection (PSGs) on target branches by codeml with the likelihood ratio test (*P* value ≤ 0.05) or a posterior probability (>0.95) in PAML.

### 2.11. Gene family expansion and contraction

In this study, the Pfam gene families were used to analyze expansion and contraction by CAFE 4.2.1.^75^ The global parameter λ, which described both the gene birth (λ) and death (μ = −λ) rate across all branches in the tree for all gene families, was estimated to be 0.00151763700272 using ML. The error model value which represented the error probability of gene family size was set to be 0.0125 through iterative computations by caferror.py in CAFE. A conditional *P* value was calculated for each gene family, and families with conditional *P* values < threshold (0.01) were considered as having an accelerated rate of gain or loss.

### 2.12. Gene family analysis

Five chemosensory gene families were focused on and identified through HMM models from Pfam A database, including GRs (PF06151 and PF08395), ORs (PF02949), IRs (PF00060), OBPs (PF01395), CSPs (PF03392). Then, we further manually annotated these genes in four fig wasp species by HMM model search (http://www.hmmer.org/, 22 May 2022, date last accessed) and Interproscan software screening.^76^ The five types of chemosensory genes of *D. melanogaster*, *A. mellifera* and *N. vitripennis* have been fully studied, so we used them as outgroup^77–81^ to construct phylogeny by ML method in FastTree v 2,^82^ and the confidence of each branch was tested by bootstrap 1,000 times. According to Orthomcl software^66^ and the cladistic clustering relationship of the phylogenetic tree, the five chemosensory gene families, OBPs, CSPs, ORs, GRs and IRs of four fig wasp species were divided into different orthologous groups.

The venom proteins of four fig wasp species and other Hymenoptera insects were also analyzed in this study. Seventy-nine venom protein sequences were reported in the model species of *N. vitripennis,*^83^^,^^84^ of which 41 protein sequences can be downloaded in NCBI. We used these 41 protein sequences to blast venom genes in four fig wasp species and other ten Hymenoptera species (including *N. vitripennis*) and *D. melanogaster*. Venom proteins belong to secretory proteins, so we retained the protein sequences with signal peptide but without transmembrane region.

In addition, three detoxification gene families were focused on and identified through HMM models from Pfam A database, including Glutathione-S-transferases (GSTs; PF00043; PF02798; PF13409; PF13410; PF13417; PF14497; PF17171), cytochrome P450s (P450s; PF00067), and carboxylesterases (CCEs; PF00135). The three types of detoxification genes of *A. mellifera* and *N. vitripennis* as outgroup to construct phylogeny by ML method in FastTree v2,^82^ and the confidence of each branch was tested by bootstrap 1,000 times.

## 3. Results and discussion

### 3.1. Chromosome-level genome assembly

The genome of *V. javana* sequenced was generated 28.98 Gb PacBio long reads and 35.27 Gb Illumina short reads, with 96.26 × and 112.79 × genome coverage respectively (Table 1 and Supplementary Table S2). Genome size was estimated to be 304.2 Mb based on 21 K-mer analysis according to Illumina reads (Supplementary Fig. S1). After genome assembly of the PacBio reads and correction with Illumina reads, we obtained a reference of 296.34 Mb with contig N50 of 26.76 Mb (Supplementary Table S3). The genome size is similar to that of three other fig wasp species, *W. pumila* (318 Mb), *E. verticillate* (387 Mb) and *C. solmsi* (277 Mb), respectively. Although the contig N50 length of *V. javana* is much longer than those of three other fig wasp species, *W. pumila* (10.9 Mb), *E. verticillate* (3.13 Mb) and *C. solmsi* (0.075 Mb) (Table 1).

**Table 1:** Assembly statistics for four Agaonidae genomes

Features	*V. javana*	*W. pumila*	*E. verticillata*	*C. solmsi*
Family	Agaonidae	Agaonidae	Agaonidae	Agaonidae
Sequencing technology	Pacbio+Illumina	Pacbio+Illumina+Hi-C	PacBio	Illumina
Genome coverage	96.26 ×+112.79 ×	—	170×	92.9×
Genome size (Mb)	296.34	318	387	277
Number of contigs	13	102	768	15,018
Contig N50 (Mp)	26.76	10.9	3.13 Mb	0.075
BUSCO genes (%)	92.7	93.3	86.8	92.7
GC content (%)	27.43	30.34	29.8	30.4
Repeat content (%)	7.30	8.14	21.94	9.30
Number of protein-coding genes	14,333	16,457	14,012	9,817

**Figure 1:**
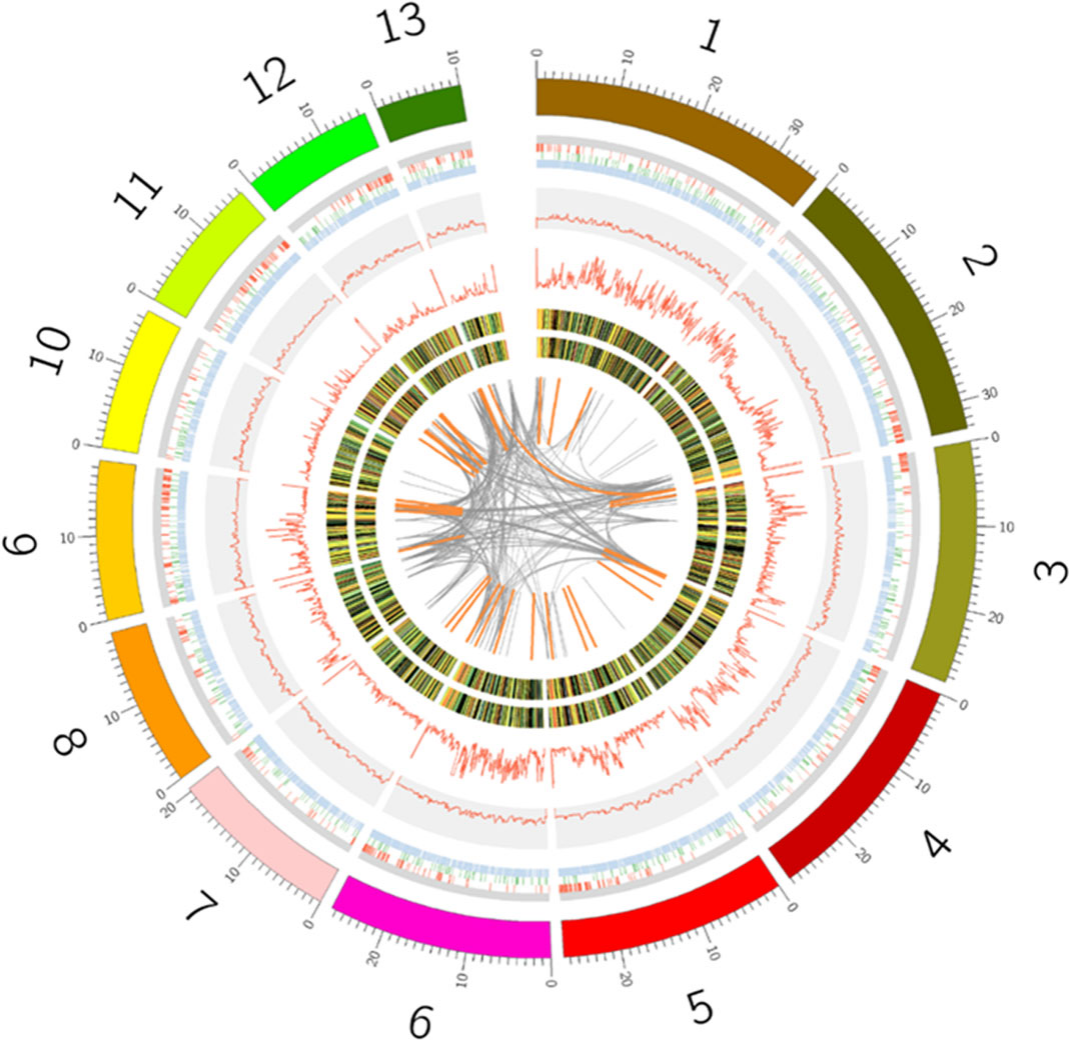
Genome landscape of the fig wasp *V. javana*. From outer to inner circles: I, 13 contigs represented 98.45% genome of *V. javana*; II, 4 classes of repeat sequences in genome with simple sequence repeat, long terminal repeats (LTR), DNA transposon and unclassified repeat from outer to inner; III, GC content across the genome, drawn in 100-kb non-overlapping windows; IV, number of single nucleotide polymorphism (SNPs) calculated in 100-kb non-overlapping windows with the max value of the axis 60; V, gene expression of transcriptome for the samples stimulated by odor (inner ring) and the controls (out ring); VI, regions sharing more than 90% sequence identity.

The assembly of *V. javana* genome is high-quality with 13 contigs assembled from genomes *de novo* (Fig. 1). We assessed the completeness of the assembly using BUSCO, and 5,516 out of 5,991 (92.1%) conserved Hymenoptera genes were found in the whole-genome nucleotide sequences of *V. javana* (Supplementary Fig. S2). In addition, we further compared the BUSCO integrity of the genome of other published fig wasp species, *W. pumila*, *E. verticillata* and *C. solmi*, and the results were all ∼92% (Supplementary Fig. S2). Although the genome assembly of *W. pumila* has reached chromosome level. This indicates that the Hymenoptera_ Odb10 provided by BUSCO is mainly constructed according to the protein sequences of some model species, such as *A.mellifera* and *Acromyrmex echinatiord*, so it may be not suitable for fig wasps.

The telomere sequence of animal genome is usually simple repeat sequence. By analyzing the genomic sequence of *V. javana*, we found there are three kinds of repeat units in telomere sequence: ATTGGGTT, ATCGGGTT and ATCTATTC (Supplementary Table S4). The first two telomeres are located at one end of the chromosome, and the third telomere is located at the other end of the chromosome. Among the 13 contigs, 7 of them have 2 telomeres at 2 ends; 4 of them were found 1 telomere at the end suggesting a good integrity of this genome assembly.

### 3.2. Genome annotation

In total, 7.14% of the *V. javana* genome consists of repeat sequences, of which transposons are 0.61%. The main repeat is simple repeats with 4.02% of the genome (Supplementary Table S5 and Fig. 1). The rate of repetitive sequences in genome of *V. javana* is similar to that of two fig wasps, *W. pumilae* and *C. solmi*, and smaller than that of most insects of the remaining Hymenoptera and other orders (Supplementary Table S6).

Non-coding genes in eukaryotes mainly include rRNA, tRNA, small nuclear RNA and miRNA. In genome of *V. javana*, we annotated 62 microRNAs, 119 tRNAs, 31 rRNAs and 30 small nuclear RNAs (Supplementary Table S7). In addition to the above four types of non-coding RNAs, 26 histone downstream element were found in the genome of *V. javana*, which is a stem-loop rich in purine in the untranslated region (UTR) region at the 3′ end of histone. By combining with stem loop binding protein, the precursor of histone mRNA was processed to form a special stem loop at the tail of 3′ end, and not the common PolyA tail.^85^

We predicted 12,797 protein-coding genes in the genome of *V. javana* based on three lines of evidence. The protein-coding gene number of *V. javana* is similar to that of most Chalcidoidea wasps which are from NCBI, such as *E. verticillata* with 14,012 genes, *N.vitripennis* (parasitic wasp: 13,589), and *C.floridanum* (parasitic wasp: 12,136 genes; Supplementary Table S8). For *V. javana*, the length of gene, cDNA and CDS were 5,462, 1,915 and 1,002 bp, respectively. The median length of single exon and single intron were 214 and 412 bp, with an average of 5 exons per gene (Supplementary Table S8).

More than 80.53% of the predicted genes (10,306 genes) of *V. javana* have homology in public databases of Nr, Swissprot, KOG, EggNOG, InterPro, KEGG and GO (Supplementary Table S9).

### 3.3. Comparative genomics and phylogenetic reconstruction

We blasted 391,324 genes by blastp among *V. javana* and the other 29 insect species used in our analysis, of which 288,079 genes (37.51%) of all 30 species could be identified as orthologous genes among at least 2 species, and were divided into 27,219 OCGs. Among these OCGS, functions of 27,011 OCGs (99.23%) could be annotated. In genome of *V. javana*, 10,051 (70.12%) genes were distributed in 9,633 OCGS, 1,210 (8.44%) were identified as paralog and 3,072 (21.42%) were orphan genes.

**Figure 2:**
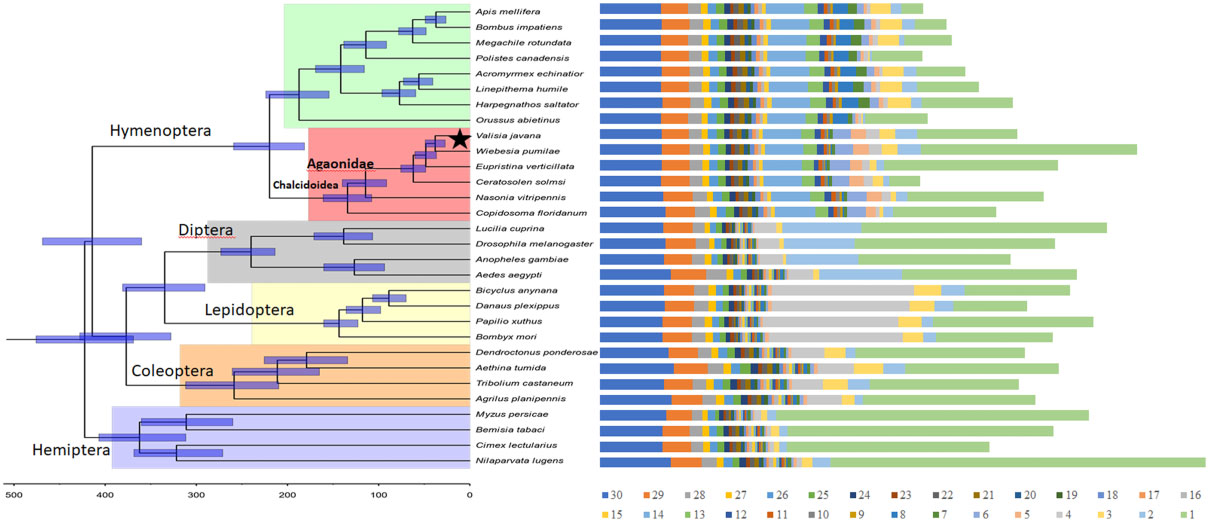
A ML phylogenetic tree shown for *V. javana* (asterisk marker) with 13 insect species in Hymenopterans (including other three fig wasp species) and the other insect species in 4 orders, each with 4 species, along with the number of common orthologous genes across 30 insect species of all their genome genes with 1 (specific) –30 classes (exists in all 30 species). The phylogenetic tree was based on 1,647 single-copy proteins. The Hemiptera species were used as the outgroup. The bootstrap value of all nodes is supported at 100/100. Divergence times are indicated by bars at the internodes, and the bars indicates the 95% confidence interval of the divergence time. The family Agaonidae, superfamily Chalcidoidea and four orders are shown on the branches.

The number of orthologous genes is from 2,448 to 2,827 across 30 insect species accounting for 14.58–25.03% of all their genome genes (Fig. 2). The number and proportion of orphan genes in different species vary greatly, whereas the number of orthologous genes in related species is very close.

The phylogenetic relationships between *V. javana* and the other 29 insect species were determined with a genome-wide set of 1,647 single-copy genes (Fig. 2). As expected, *V. javana* has a closer relationship to three other fig wasp species than the other two species of chalcidoids. The six chalcidoids (*V. javana*, *W. pumila*, *E. verticillata*, *C. solmsi*, *N. vitripennis* and *C. floridanum*) cluster together, with eight other Hymenoptera insects as a sister group. Estimated divergence times of *V. javana* and *W. pumila* (calculated using mcmctree) suggest that *V. javana* diverged from the common ancestor of the other members in the family Agaonidae ∼39.22 Mya (28.1–50.7 Mya; Fig. 2). The lineage to which *N. vitripennis* belongs was estimated to have diverged from *C.floridanum* ∼120Mya, while 113 Mya was estimated for the divergence of the clade including *N.vitripennis* and *C.solmsi* from *C.floridanum* in.^86^

### 3.4. Genome synteny

**Figure 3:**
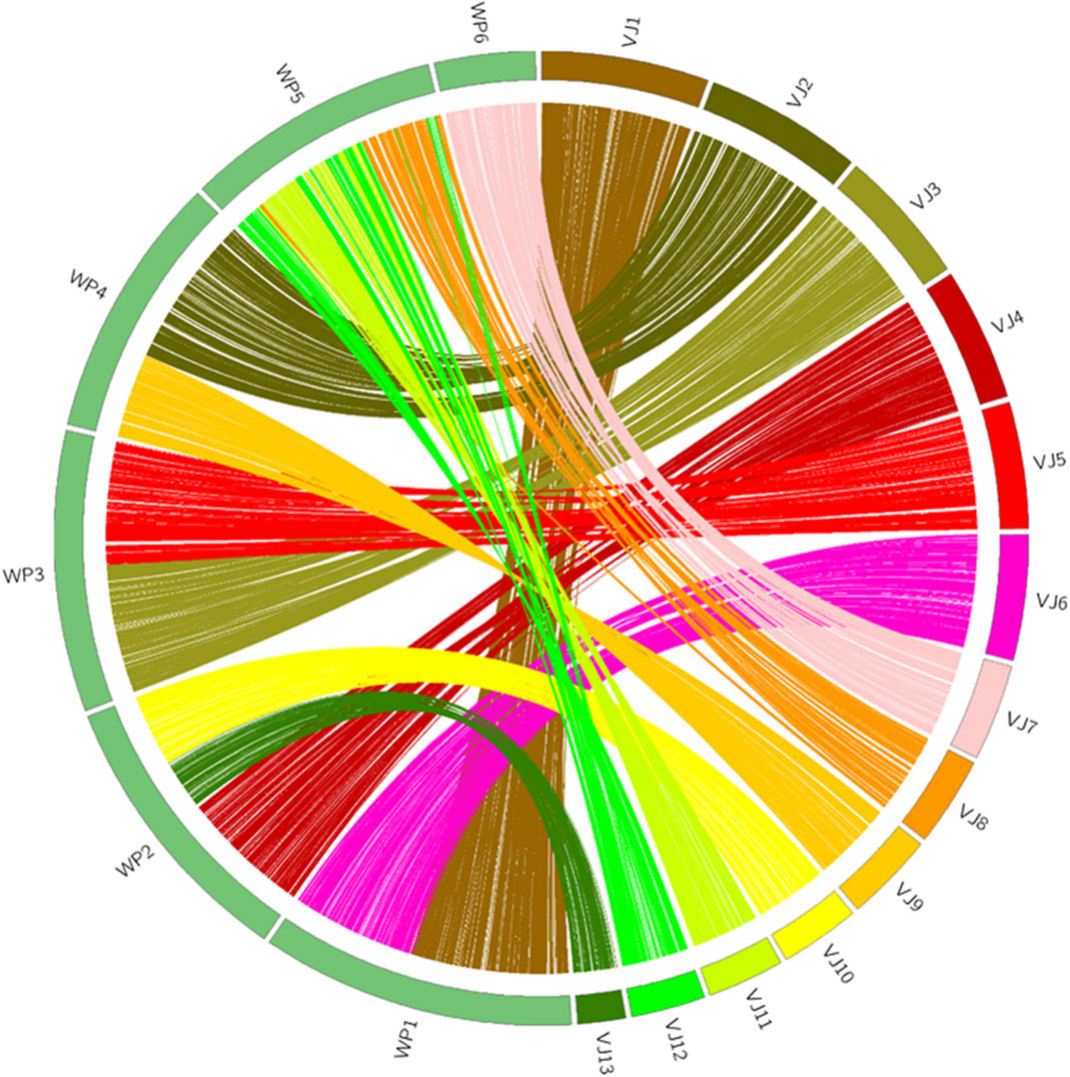
Synteny between genomes of *V. javana* and *W. pumila.* Colored lines indicate homologous genes shared syntenic blocks (containing at least 10 orthologous genes).

According to the topological structure of the phylogenetic tree, the evolutionary relationship between *V. javana* and *W. pumila* is the closest. So, synteny of the *V. javana* assembly was compared with *W. pumila*. The synteny analysis is based on the same distributed sequence of orthologous genes between two species. *V. javana* showed a high level of synteny with *W. pumila* which was consistent with their closer relationship in the phylogenetic tree (Fig. 3).

There are 8,800 genes (59.30% of the genome) in *V. javana* and 8,612 genes in *W. pumila* distributed in 168 synteny blocks, of which 53 belong to large synteny blocks (containing ≥ 50 homologous genes), of which the largest block contains 293 homologous genes. For *V. javana*, three groups of contig 1 and contig 6, contig 2 and contig 9, contig 3 and contig 5 are matched to Chromosomes 1, 4, 3 of *W. pumila,* respectively.

### 3.5. Genes under positive selection

A total of 27 and 25 PSGs were identified in *V. javana* and Agaonidae respectively from the 66 candidate positive selection genes (Supplementary Table S10). There are 14 PSGs shared between the clade of *V. javana* and Agaonidae, which are mainly related to signal transduction, genetic information processing, nervous system and endocrine system. For *V. javana*, the most significant PSGs (*P* = 0) were innexin, LIM domain transcription factor and LIM domain protein. Innexin belongs to hexameric protein family and can constitute gap junctions to perform the function of intercellular communication.^87^ LIM domain transcription factor and LIM domain protein play an important role in the development and regulation of perception and movement.^88^ In Agaonidae, there is one PSG, pikachrin, which can help transmit electrical signals from retina to brain more quickly and efficiently,^89^ and maybe related to the light induction of fig wasps to the open of emission hole on syconium wall and during flight.

### 3.6. Gene family expansion and contraction

When compared with *W. pumila*, 8 and 10 gene families were expanded and contracted in the *V. javana* genome according to Pfam blasting (Supplementary Table S11). Among the expanded Pfam, there are OR, OBP, trypsin (PF00089), Kazal-type serine protease inhibitor domain (PF00050, PF07648), G protein-coupled chemoreceptor protein (PF10328) and transcription factor (PF13909). The expansion of OR, OBP and G protein coupled chemoreceptor protein suggests that *V. javana* may have stronger odor perception than *W. pumila*. Trypsin is an important digestive protein in most insects, which is widely distributed in the digestive tract of insects of different orders and feeding habits.^90^ The number of serine protease inhibitors in the genome of *V. javana* is as high as 235, which can inhibit the activity of serine protease and has antibacterial activity.^91^ Serine protease inhibitor may exist in the venom protein of insects,^92^ and protect their eggs.^93^ The contraction gene family in *V. javana* is mainly histone gene family: core histone H2A/H2B/H3/H4 (PF00125); histone-like transcription factor (CBF/NF-Y) and archaeal histone (PF00808); C-terminus of histone H2A (PF16211; Supplementary Table S11).

When compared with *N. vitripennis*, 2 and 82 gene families were expanded and contracted in the clade of Agaonidae which contained four fig wasp species according to Pfam blasting (Supplementary Table S11). Two expanded gene families are pao retrotransposon peptidase and trypsin. Among the contracted gene families, several are chemosensory genes (e.g. CSP, OBP, OR), detoxification genes (e.g. P450, CCE, ecdysteroid kinase-like), ANK, histone-related genes and cuticle protein which are major components of the insect cuticle-associated organs such as integument and wings.^94^

### 3.7. Deg of *V. javana* between odor treatment and controls

In total, 22.71–30.03 M read pairs were obtained from 6 samples of odor stimulated and the control for *V. javana* transcriptome, respectively. After quality control, 91.85–96.32% of reads pairs of data were left and 96.47–97.66% of them can be matched to its genome (Supplementary Table S12 and Fig. 1).

**Figure 4:**
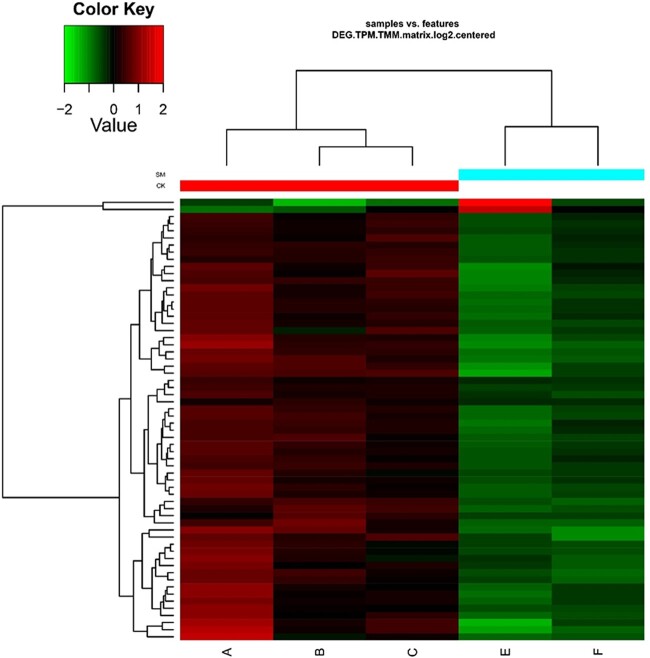
Heatmap of DEGs in transcriptome of *V. javana* between odor-stimulated samples and the controls. The samples from A to C are the controls, whereas E and F are odor-stimulated. Sample D was removed because it was quite different from the other two biological replicates during correlation analysis.

There were two genes were up-regulated and 60 genes were down-regulated after olfactory treatment (Fig. 4). The up-regulated genes are venom gene CCE and the unknown functional gene, respectively. Go and KEGG pathway enrichment showed down-regulated genes played an important role in signal transduction.

### 3.8. Chemosensory genes

**Figure 5:**
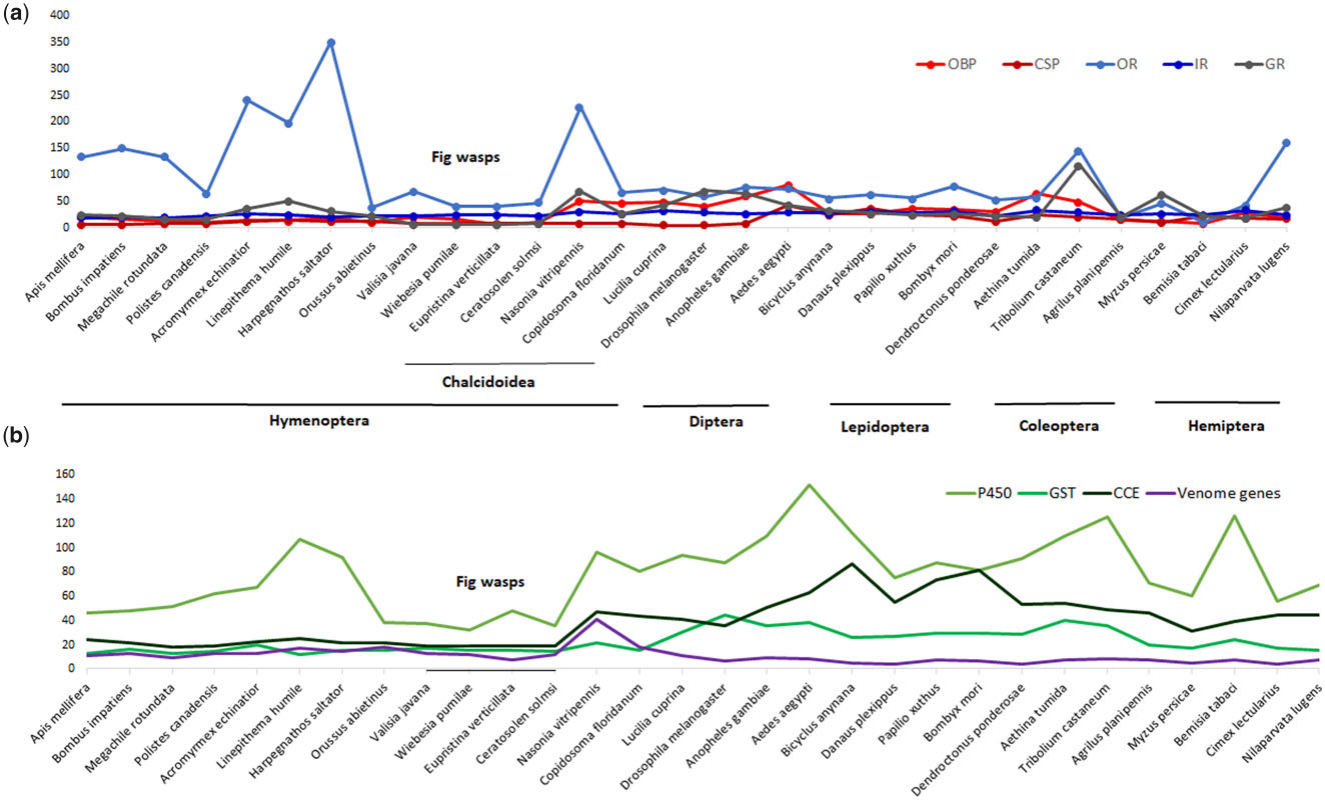
The number of chemosensory, detoxification and venom gene families among 30 insect species (nodes represent the number of genes). (a) Chemosensory genes, OBP, CSP, OR, IR and GR for each species. (b) Detoxification genes, P450s, GSTs, and CCEs and venom genes for each species.

Five chemosensory gene families were blasted by Pfam in 30 species genomes (Fig. 5a and Supplementary Table S13). We further identified these genes by manual annotation in four fig wasp species. The number of them in each family is the same as that obtained by Pfam except for ORs with one less in each of the three species, *V. javana*, *W. pumila* and *C. solmsi*, which implies that the genes got by blasting Pfam are reliable. OBPs are involved in the first step of olfactory signal transduction, carrying airborne semi-chemicals to the ORs.^95^ The number of OBPs in fig wasps is smaller than those in two other wasp species in the same super-family of Chalcidoidea, *N. vitripennis* and *C. floridanum* who are both semi-generalist parasitic wasp,^96^^,97^ which may relate to the relatively simple and closed living environment of fig host.^28^ The number of OBPs in four fig wasp species is from 6 to 20 (Supplementary Table S13), and divided into more than 17 groups, of which only 4 groups (1–4) contained genes of all four fig wasp species (Supplementary Fig. S3). Groups 2, 3 and 14 were clustered into one clade with Dmelobp83a and Dmelobp83b from *D. melanogaster* which are responsible for the detection of pheromones.^77^^,^^98^ Group 5 and 17 are related and contain five repeats OBPs with three of them highly expressed in *V. javana* transcriptome (Supplementary Fig. S3 and Table S14). These two groups clustered into one clade with DmelOBP69a, which has been predicted binding phenylalkylamine and pheromone involved both courtship and olfactory behavior^77^^,^^98^^,99^ and may have important functions in fig wasps. According to *V. javana* transcriptome, one OBP gene was highly expressed in Groups 7, 8, 9 and 12, respectively (Supplementary Table S14). These four groups clustered into one clade with DmelOBP56a, DmelOBP56d and DmelOBP56e which can bind to odor compound or pheromones.^77^

CSPs is much more conserved across fig wasps, with fewer quantity differences among species and more homology groups contained genes from all four fig wasps (Fig. 5a, Supplementary Table S13 and Fig. S4). In total, there are eight CSPs for each of fig wasp species and divided into eight groups contained genes from all four fig wasps. Groups 1, 4, 5 and 7 can be clustered into one clade with CSPs of *A. mellifera* (Supplementary Fig. S4). Four CSPs with high expression in *V. javana* transcriptome and two of them in Groups 1 and 7 clustered with AmelCSP1 and AmelCSP5/AmelCSP2 respectively (Supplementary Fig. S4 and Table S14). AmelCSP1 has strong affinity to straight chain alcohols and esters, whereas AmelCSP2 has strong binding ability to aromatic compounds.^79^

The number of ORs in fig wasp species was usually smaller than other insects in Hymenoptera (Fig. 5a and Supplementary Table S13). When compared with other chemosensory genes, ORs of fig wasps can be divided into more groups showing their olfactory sense system is more complex and olfactory recognition is more extensive (Supplementary Fig. S5). They can not only identify the subtle differences of volatile chemicals released by different fig species, but also facilitate the complex information exchange between fig wasps. Meanwhile, fewer groups can be clustered into one clade with ORs of *D. melanogaster* means fig wasp species has stronger specificity and faster evolution rate. Group 1 in ORs tree of fig wasps was clustered into one clade with Dmelor83b of *D. melanogaster*, which is a kind of co-receptor (Orco) remains in a complex with ORs in the sensory compartment to enhance the sensitivity of common ORs to the corresponding odor molecules.^100^ In Group 2, we found one OR gene having eight repeats in *V. javana*, whereas in Group 3 there is also one OR gene having two repeats and having four repeats in *C. solmsi* (Supplementary Fig. S5). The serial numbers of most repeats are consecutive which imply they may gained by tandem gene duplications.^101^ So, the number of OR in both species is a little higher than that of the other two species, *W. pumilae* and *E. verticillate*. We also found some ORs which have a relatively high expression in *V. javana* transcriptome (Supplementary Fig. S5 and Table S14).

The number of IRs varied little among insect species (Fig. 5a and Supplementary Table S13). IRs in fig wasp species can be divided into 22 groups in which several can be clustered with IRs of *A. mellifera* and *D. melanogaster* (Supplementary Fig. S6). The genes of Groups 7 and 8 in fig wasp IR gene tree converged with IR25a and IR8a from both *A. mellifera* and *D. melanogaster*, respectively (Supplementary Fig. S6). IR8a and IR25a are two kind of co-receptor and keep high sequence similarity among insect species.^102^^,103^ Five IRs were highly expressed in transcriptome of *V. javana* in five different groups, and none of them were in the same branch as the related genes of two model species.

We found more GRs in three fig wasp species than their published genomes according to Pfam codes PF06151 and PF08395 (Fig. 5a and Supplementary Table S13). GRs of fig wasps can be divided into several groups, of which only two groups (1 and 2) contained genes of all four fig wasp species and converged with DmelGR64e, DmelGR64f and DmelGR5a, which mainly involved in the detection of sweet chemicals (Supplementary Fig. S7).^80^^,^^81^^,^^104^ The adults of fig wasps do not need to eat, and the other life cycles, such as egg, larva and pupa stage are all spent in the host’s syconium and feed on the endosperm formed by galls enlargement.^105^^,106^ Therefore, the GRs of fig wasps should be mainly sensitive to sugars.

### 3.9. Venom genes

When compared with 41 venom genes in *N. vitripennis* downloaded from NCBI, we found 13, 12, 7 and 12 venom genes in *V. javana*, *W. pumila*, *E. verticillata* and *C. solmi,* respectively and divided into 10 functional categories (Fig 5b, Supplementary Tables S15 and S16) including proteases and peptidases, protease inhibitors, carbohydrate metabolism, DNA metabolism, glutathione metabolism, esterases, recognition/binding proteins, immune-related proteins, others and unknown.^84^ The number of venom genes of four fig wasp species is far less than that of *N. vitripennis*, which may due to *N. vitripennis* semi-generalists parasitic while fig wasps highly species-specific.^96^^,97^ From the annotated venom genes, three are present in all four species of fig wasps and *N. vitripennis*: chitinase 5 of carbohydrate metabolism, acid phosphatase and arylsulphatase b of esterases that may alter host physiology to support the developments of endoparasitoid by cellular degradation, cell signaling, hormone regulation or by inhibiting synthesis of certain proteins.^107^^,108^

### 3.10. Detoxification genes

The genes of GSTs, P450s and CCEs are mainly involved in the detoxification and metabolism of compounds by insects.^109–111^ In addition, CCE also plays a key role in insect development and behavior, and participates in the degradation of odor molecules and chemical pheromones, insect reproduction and digestion.^112^ P450 has a wide range of substrates and diverse catalytic functions, and can also participate in a series of physiological and biochemical reactions in insects except detoxification, such as the regulation of hormones, steroids and fatty acids.^113^^,114^

When compared with *N. vitripennis*, we observed some shared events of gene losses among four fig wasp species, such as Clades 2, 3, 4, 5 and 7 in P450s family (Supplementary Fig. S8), Clade 2 in GSTs family (Supplementary Fig. S9) and three clades in CCE family (Supplementary Fig. S10), possibly attributable to ancestral divergence from other Chalcidoidea insects. The reductions of three gene families in fig wasps may be related to its strict host specificity (Fig. 5b) and thus most of its lifetime spending in a closed inflorescence.^28^ Although the fewer number gene of three detoxification enzymes in *A. mellifera* has been assumed relating with its specialized eusocial behavior and homeostasis of the nest environment.^115^

### 3.11. Olfaction-related pathways

**Figure 6:**
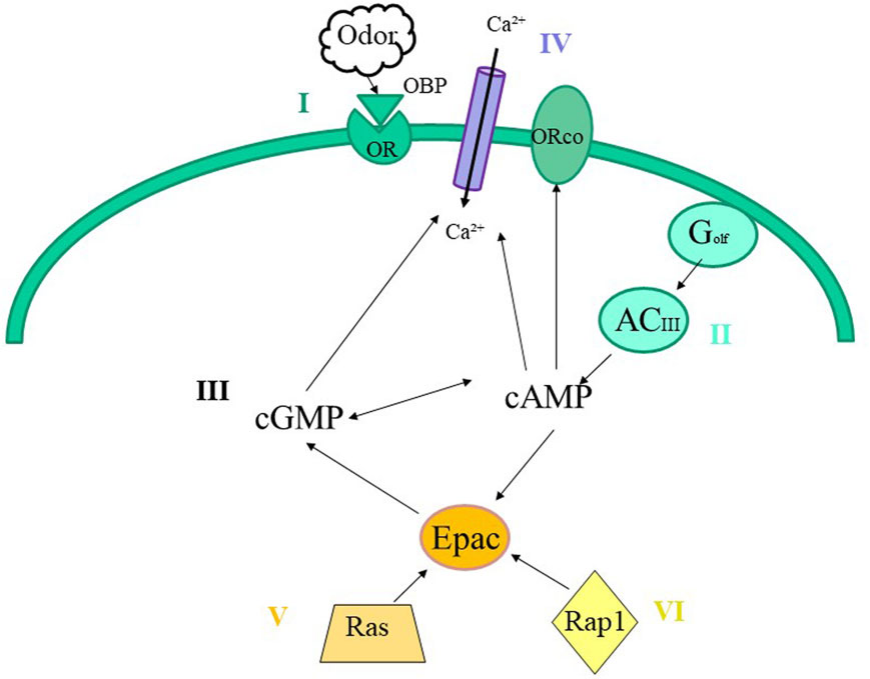
Six main pathways maybe related to olfactory signal conduction in *V. javana* with DEGs according to the transcriptome data between samples stimulated odors and the controls. The six main pathways are I, olfactory transduction; II, cAMP; III, cGMP-PKG; IV, Calcim; V, Ras; and VI, Rap1 signal transduction pathways.

From genome and comparative transcriptome sequencing, we found several DEGs between fig wasp samples stimulated by host odor and the controls, such as adenylyl cyclase, TIAM1, PLCE, adenylate cyclase and CaN, which may play important roles to regulate signal transduction.^116–118^ in cAMP, cGMP-PKG, Calcim, Ras and Rap1 signal transduction pathways which were believed to be involved in olfactory sensory transduction in both vertebrates and insects (Fig. 6 and Supplementary Figs. S11–S16).^9^^,^^119^^,120^ In insects, the exact olfactory signal transduction pathway is still elusive and must be highly asymmetric, as has also been demonstrated for vertebrate olfactory.^17^^,^^121^^,^^122^ Except the second messengers, the complex between OR and Orco in insects can confer channel activity^16^ (Fig. 6).

Most of these genes are down-regulated in odor stimulation samples than the control. The reason is that in contrast to vertebrates, invertebrates have both excitatory and inhibitory responses to odors.^123^^,^^124^ The response of insects to odor is a rapid process, while our fig wasp samples were exposed to odor for at least half an hour. Therefore, these down-regulated genes should be an inhibitory response to persistent odor stimulation. In order to have a detailed understanding of the induction and transduction of odor stimulation in fig wasps, we should give different periods of odor stimulation to fig wasps, and then compare the expression of these genes. In addition, transcriptome analysis focusing on the specialized olfactory organs (such as antennae) can find more DEGs.

## 4. Conclusions

We have provided a high-quality chromosome-level genome assembly of *V.javana* using a combined Illumina + PacBio assembly strategy. Basic genomic analyses revealed genome features, phylogenetic position, gene gains and loss and gene evolution of *V. javana*. We found chemosensory genes in four fig wasp species and compared the number, genetic relationship and try to predict some functions of them according to the predicted function of these genes in *A.mellifera* and *D.melanogaster*. We also found several DEGs in transcriptome data of *V. javana*, which play important roles in some olfaction related pathways. This genome assembly will serve as a useful resource for further research into insect biology, pollinator–host interactions, comparative genomics and coevolution.

## Supplementary data

Supplementary data are available at DNARES online.

## Funding

This work was supported by the National Natural Science Foundation of China (Grant numbers: 31971568, 32150410364 and 31630008), Province Natural Science Foundation of Guangdong (Grant number: c20140500001306) and The Chinese Academy of Sciences PIFI Fellowship for Visiting Scientists (2022VBA0002).

## Conflict of interest

None declared.

## Data availability

All genome sequence data of *V. javana* are available in GWH database of the National Genomics Data Center (NGDC) with accession no. GWHBDGE00000000. The raw genome sequencing data of Illumina and PacBio have deposited in the GSA database of the National Genomics Data Center (NGDC) with accession number of CRA004469. The *V. javana* transcriptome data are available in the GSA database of the National Genomics Data Center (NGDC) with accession number CRA004554.

## Supplementary Material

dsac014_Supplementary_Data

## References

[1] Thompson J.N. 1994, The Coevolutionary Process. Chicago: University of Chicago.

[2] Thompson J.N. 2005, The Geographic Mosaic of Coevolution. Chicago: University of Chicago.

[3] Bronstein J.L. , Alarcón R., Geber M. 2006, The evolution of plant-insect mutualisms, New Phytol., 172, 412–28. 17083673 10.1111/j.1469-8137.2006.01864.x

[4] Weiblen G.D. 2002, How to be a fig wasp, Annu. Rev. Entomol., 47, 299–330. 11729077 10.1146/annurev.ento.47.091201.145213

[5] Rønsted N. , Weiblen G.D., Cook J.M., Salamin N., Machado C.A., Savolainen V. 2005, 60 million years of co-divergence in the fig-wasp symbiosis, Proc. Roy. Soc. Lond. B, 272, 2593–9. 10.1098/rspb.2005.3249 PMC1559977 16321781

[6] Yu H. , Tian E., Zheng L., et al 2019, Multiple parapatric pollinators have radiated across a continental fig tree displaying clinal genetic variation, Mol. Ecol., 28, 2391–405. 30753744 10.1111/mec.15046

[7] Berg C.C. , Wiebes J.T. 1992, African Fig Trees and Fig Wasps. Amsterdam: Royal Netherlands Academy of Arts & Sciences.

[8] Janzen D.H. 1979, How to be a fig, Annu. Rev. Ecol. Syst., 10, 13–51.

[9] Ache B.W. , Young J.M. 2005, Olfaction: diverse species, conserved principles, Neuron, 48, 417–30. 16269360 10.1016/j.neuron.2005.10.022

[10] Hossaert-Mckey M. , Soler C., Schatz B., Proffit M. 2010, Floral scents: their roles in nursery pollination mutualisms, Chemoecology, 20, 75–88.

[11] Wang R. , Yang Y., Jing Y., et al 2021, Molecular mechanisms of mutualistic and antagonistic interactions in a plant-pollinator association, Nat. Ecol. Evol., 5, 974–86. 34002050 10.1038/s41559-021-01469-1

[12] Grison L. , Edwards A.A., Hossaert-McKey M. 1999, Interspecies variation in floral fragrances emitted by tropical *Ficus* species, Phytochemistry, 52, 1293–9.

[13] Proffit M. , Chen C., Soler C., Bessière J.M., Schatz B., Hossaert-McKey M. 2009, Can chemical signals responsible for mutualistic partner encounter promote the specific exploitation of nursery pollination mutualisms? The case of figs and fig wasps, Entomol. Exp. Appl., 131, 46–57.

[14] Ware A.B. , Kaye P.T., Compton S.G., Noort S.V. 1993, Fig volatiles: their role in attracting pollinators and maintaining pollinator specificity, Plant Syst. Evol., 186, 147–56.

[15] Hildebrand J.G. , Shepherd G.M. 1997, Mechanisms of olfactory discrimination: converging evidence for common principles across phyla, Annu. Rev. Neurosci., 20, 595–631. 9056726 10.1146/annurev.neuro.20.1.595

[16] Sato K. , Pellegrino M., Nakagawa T., Nakagawa T., Vosshall L.B., Touhara K. 2008, Insect olfactory receptors are heteromeric ligand-gate ion channels, Nature, 452, 1002–7. 18408712 10.1038/nature06850

[17] Nakagawa T. , Vosshall L.B. 2009, Controversy and consensus: noncanonical signaling mechanisms in the insect olfactory system, Curr. Opin. Neurobiol., 19, 284–92. 19660933 10.1016/j.conb.2009.07.015 PMC2752668

[18] Danneels E.L. , Rivers D.B., Graaf D., Dirk C. 2010, Venom proteins of the parasitoid wasp *Nasonia vitripennis*: recent discovery of an untapped Pharmacopee, Toxins (Basel), 2, 494–516. 22069597 10.3390/toxins2040494 PMC3153221

[19] Moreau S.J.M. , Asgari S. 2015, Venom proteins from parasitoid wasps and their biological functions, Toxins (Basel), 7, 2385–412. 26131769 10.3390/toxins7072385 PMC4516919

[20] Lin Z. , Wang R.-J., Cheng Y., et al 2019, Insights into the venom protein components of Microplitis mediator, an endoparasitoid wasp, Insect Biochem. Mol. Biol., 105, 33–42. 30602123 10.1016/j.ibmb.2018.12.013

[21] Galil J. , Eisikowitch D. 1968, Flowering cycles and fruit types of *Ficus sycomorus* in Israel, New Phytol., 67, 745–58.

[22] Ramirez W.B. 1969, Fig wasps: mechanism of pollen transfer, Science, 163, 580–1. 17750894 10.1126/science.163.3867.580

[23] Gauld I. , Bolton B. 1988, The Hymenoptera. Oxford: Oxford University Press in association with British Museum (Natural History).

[24] Martinson E.O. , Jandér K.C., Peng Y.-Q., et al 2014, Relative investment in egg load and poison sac in fig wasps: implications for physiological mechanisms underlying seed and wasp production in figs, Acta Oecol., 57, 58–66.

[25] Tellier A. , Moreno-Gamez S., Stephan W. 2014, Speed of adaptation and genomic footprints of host-parasite coevolution under arms race and trench warfare dynamics, Evolution, 68, 2211–24. 24749791 10.1111/evo.12427

[26] Werren J.H. , Richards S., Desjardins C.A., et al 2010, Functional and evolutionary insights from the genomes of three parasitoid Nasonia species, Science, 327, 343–8. 20075255 10.1126/science.1178028 PMC2849982

[27] Yin C. , Li M., Hu J., et al 2018, The genomic features of parasitism, polyembryony and immune evasion in the endoparasitic wasp *Macrocentrus cingulum*, BMC Genom., 19, 420. 10.1186/s12864-018-4783-x PMC5977540 29848290

[28] Xiao J.H. , Yue Z., Jia L.Y., et al 2013, Obligate mutualism within a host drives the extreme specialization of a fig wasp genome, Genome Biol., 14, R141–18. 24359812 10.1186/gb-2013-14-12-r141 PMC4053974

[29] Zhang X.T. , Wang G., Zhang S.C., et al 2020, Genomes of the banyan tree and pollinator wasp provide insights into fig-wasp coevolution, Cell, 183, 875. 33035453 10.1016/j.cell.2020.09.043

[30] Wang G. , Zhang X.T., Herre E.A., et al 2021, Genomic evidence of prevalent hybridization throughout the evolutionary history of the fig-wasp pollination mutualism, Nat. Commun., 12, 718 33531484 10.1038/s41467-021-20957-3 PMC7854680

[31] Berg C.C. 2003, Flora Malesiana precursor for the treatment of Moraceae 1: the main subdivision of *Ficus*: the subgenera, Blumea, 48, 167–78.

[32] Yu H. , Zhao N.X., Chen Y.Z., Herre E.A. 2008, Male and female reproductive success in the dioecious fig, *Ficus hirta* Vahl, in Guangdong Province, China: implications for the relative stability of dioecy and monoecy, Symbiosis, 45, 121–8.

[33] Tian E.W. , Yu H., Zhang D.Y., Nason J.D. 2011, Development of microsatellite loci for *Blastophaga javana* (Agaonidae), the pollinating wasp of *Ficus hirta* (Moraceae), Am. J. Bot., 98, e41–3. 21613105 10.3732/ajb.1000432

[34] Tian E.W. , Nason J.D., Machado C.A., Zheng L.N., Yu H., Kjellberg F. 2015, Lack of genetic isolation by distance, similar genetic structuring but different demographic histories in a fig pollinating wasp mutualism, Mol. Ecol., 24, 5976–91. 26518361 10.1111/mec.13438

[35] Bolger A.M. , Lohse M., Usadel B. 2014, Trimmomatic: a flexible trimmer for Illumina sequence data, Bioinformatics, 30, 2114–20. 24695404 10.1093/bioinformatics/btu170 PMC4103590

[36] Koren S. , Walenz B.P., Berlin K., Miller J.R., Bergman N.H., Phillippy A.M. 2017, Canu: scalable and accurate long-read assembly via adaptive k-mer weighting and repeat separation, Genome Res., 27, 722–36. 28298431 10.1101/gr.215087.116 PMC5411767

[37] Chin C.S. , Peluso P., Sedlazeck F.J., et al 2016, Phased diploid genome assembly with single-molecule real-time sequencing, Nat. Methods, 13, 1050–4. 27749838 10.1038/nmeth.4035 PMC5503144

[38] Ye C. , Hill C.M., Wu S., Ruan J., Ma Z.S. 2016, DBG2OLC: efficient assembly of large genomes using long erroneous reads of the third generation sequencing technologies, Sci. Rep., 6, 2014. 10.1038/srep31900 PMC5004134 27573208

[39] Guan D. , McCarthy S.A., Wood J., Howe K., Wang Y., Durbin R. 2020, Identifying and removing haplotypic duplication in primary genome assemblies, Bioinformatics, 36, 2896–8. 31971576 10.1093/bioinformatics/btaa025 PMC7203741

[40] Hu J. , Fan J., Sun Z., Liu S. 2020, NextPolish: a fast and efficient genome polishing tool for long-read assembly, Bioinformatics, 36, 2253–5. 31778144 10.1093/bioinformatics/btz891

[41] Ranallo-Benavidez T.R. , Jaron K.S., Schatz M.C. 2020, GenomeScope 2.0 and Smudgeplot for reference-free profiling of polyploid genomes, Nat. Commun., 11, 1432. 32188846 10.1038/s41467-020-14998-3 PMC7080791

[42] Seppey M. , Manni M., Zdobnov E.M. 2019, BUSCO: assessing genome assembly and annotation completeness, in Kollmar M., ed. Gene Prediction: Methods and Protocols, 227–45. New York, NY: Springer New York. 10.1007/978-1-4939-9173-0_14 31020564

[43] Langmead B. , Salzberg S.L. 2012, Fast gapped-read alignment with Bowtie 2, Nat. Methods., 9, 357–59. 22388286 10.1038/nmeth.1923 PMC3322381

[44] Flynn J.M. , Hubley R., Goubert C., et al 2020, RepeatModeler2 for automated genomic discovery of transposable element families, Proc. Natl. Acad. Sci. U. S. A., 117, 9451–7. 32300014 10.1073/pnas.1921046117 PMC7196820

[45] Tarailo-Graovac M. , Chen N.S. 2009, Using repeatmasker to identify repetitive elements in genomic sequences, Curr. Protoc. Bioinform., 25, 4.10.1–14. 10.1002/0471250953.bi0410s25 19274634

[46] Bao W. , Kojima K.K., Kohany O. 2015, Repbase Update, a database of repetitive elements in eukaryotic genomes, Mob. DNA, 6, 11. 26045719 10.1186/s13100-015-0041-9 PMC4455052

[47] Kalvari I. , Nawrocki E.P., Ontiveros-Palacios N., et al 2021, Rfam 14: expanded coverage of metagenomic, viral and microRNA families, Nucleic Acids Res., 49, D192–200. 33211869 10.1093/nar/gkaa1047 PMC7779021

[48] Nawrocki E.P. , Eddy S.R. 2013, Infernal 1.1: 100-fold faster RNA homology searches, Bioinformatics, 29, 2933–5. 24008419 10.1093/bioinformatics/btt509 PMC3810854

[49] Chan P.P. , Lowe T.M. 2019, tRNAscan-SE: searching for tRNA Genes in Genomic Sequences, in Kollmar M., ed. Gene Prediction: Methods and Protocols, 1–14. New York, NY: Springer New York. 10.1007/978-1-4939-9173-0_1 PMC6768409 31020551

[50] Kim D. , Paggi J.M., Park C., Bennett C., Salzberg S.L. 2019, Graph-based genome alignment and genotyping with HISAT2 and HISAT-genotype, Nat. Biotechnol., 37, 907–15. 31375807 10.1038/s41587-019-0201-4 PMC7605509

[51] Birney E. , Clamp M., Durbin R. 2004, Genewise and genomewise, Genome Res., 14, 988–95. 15123596 10.1101/gr.1865504 PMC479130

[52] Stanke M. , Morgenstern B. 2005, AUGUSTUS: a web server for gene prediction in eukaryotes that allows user-defined constraints, Nucleic Acids Res., 33, W465–7. 15980513 10.1093/nar/gki458 PMC1160219

[53] Huerta-Cepas J. , Szklarczyk D., Heller D., et al 2019, eggNOG 5.0: a hierarchical, functionally and phylogenetically annotated orthology resource based on 5090 organisms and 2502 viruses, Nucleic Acids Res., 47, D309–14. 30418610 10.1093/nar/gky1085 PMC6324079

[54] Coordinators N.R. 2018, Database resources of the national center for biotechnology information, Nucleic Acids Res., 46, D8–13. 29140470 10.1093/nar/gkx1095 PMC5753372

[55] Bairoch A. , Boeckmann B., Ferro S., Gasteiger E. 2004, Swiss-Prot: juggling between evolution and stability, Brief. Bioinform., 5, 39–55. 15153305 10.1093/bib/5.1.39

[56] Tatusov R.L. , Fedorova N.D., Jackson J.D., et al 2003, The COG database: an updated version includes eukaryotes, BMC Bioinformatics, 4, 41. 12969510 10.1186/1471-2105-4-41 PMC222959

[57] Buchfink B. , Xie C., Huson D.H. 2015, Fast and sensitive protein alignment using DIAMOND, Nat. Methods., 12, 59–60. 25402007 10.1038/nmeth.3176

[58] Blum M. , Chang H.Y., Chuguransky S., et al 2021, The InterPro protein families and domains database: 20 years on, Nucleic Acids Res., 49, D344–54. 33156333 10.1093/nar/gkaa977 PMC7778928

[59] El-Gebali S. , Mistry J., Bateman A., et al 2019, The Pfam protein families database in 2019, Nucleic Acids Res., 47, D427–32. 30357350 10.1093/nar/gky995 PMC6324024

[60] Mistry J. , Finn R.D., Eddy S.R., Bateman A., Punta M. 2013, Challenges in homology search: HMMER3 and convergent evolution of coiled-coil regions, Nucleic Acids Res., 41, e121. 23598997 10.1093/nar/gkt263 PMC3695513

[61] Moriya Y. , Itoh M., Okuda S., Yoshizawa A.C., Kanehisa M. 2007, KAAS: an automatic genome annotation and pathway reconstruction server, Nucleic Acids Res., 35, W182–85. 17526522 10.1093/nar/gkm321 PMC1933193

[62] Anders S. , Pyl P.T., Huber W. 2015, HTSeq-a Python framework to work with high-throughput sequencing data, Bioinformatics, 31, 166–9. 25260700 10.1093/bioinformatics/btu638 PMC4287950

[63] Robinson M.D. , Oshlack A. 2010, A scaling normalization method for differential expression analysis of RNA-seq data, Genome Biol., 11, R25. 20196867 10.1186/gb-2010-11-3-r25 PMC2864565

[64] Robinson M.D. , McCarthy D.J., Smyth G.K. 2010, edgeR: a Bioconductor package for differential expression analysis of digital gene expression data, Bioinformatics, 26, 139–40. 19910308 10.1093/bioinformatics/btp616 PMC2796818

[65] Love M.I. , Huber W., Anders S. 2014, Moderated estimation of fold change and dispersion for RNA-seq data with DESeq2, Genome Biol., 15, 550. 25516281 10.1186/s13059-014-0550-8 PMC4302049

[66] Li L. , Stoeckert C.J., Roos D.S. 2003, OrthoMCL: identification of ortholog groups for Eukaryotic genomes, Genome Res., 13, 2178–89. 12952885 10.1101/gr.1224503 PMC403725

[67] Enright A.J. , van Dongen S., Ouzounis C.A. 2002, An efficient algorithm for large-scale detection of protein families, Nucleic Acids Res., 30, 1575–84. 11917018 10.1093/nar/30.7.1575 PMC101833

[68] Katoh K. , Standley D.M. 2013, MAFFT multiple sequence alignment software version 7: improvements in performance and usability, Mol. Biol. Evol., 30, 772–80. 23329690 10.1093/molbev/mst010 PMC3603318

[69] Castresana J. 2000, Selection of conserved blocks from multiple alignments for their use in phylogenetic analysis, Mol. Biol. Evol., 17, 540–52. 10742046 10.1093/oxfordjournals.molbev.a026334

[70] Stamatakis A. 2014, RAxML Version 8: a tool for phylogenetic analysis and post-analysis of large phylogenies, Bioinformatics, 30, 1312–3. 24451623 10.1093/bioinformatics/btu033 PMC3998144

[71] Yang Z. 2007, PAML 4: phylogenetic analysis by maximum likelihood, Mol. Biol. Evol., 24, 1586–91. 17483113 10.1093/molbev/msm088

[72] Kumar S. , Stecher G., Suleski M., Hedges S.B. 2017, TimeTree: a resource for timelines, timetrees, and divergence times, Mol. Biol. Evol., 34, 1812–9. 28387841 10.1093/molbev/msx116

[73] Wang Y. , Tang H., Debarry J.D., et al 2012, MCScanX: a toolkit for detection and evolutionary analysis of gene synteny and collinearity, Nucleic Acids Res., 40, e49. 22217600 10.1093/nar/gkr1293 PMC3326336

[74] Krzywinski M.I. , Schein J.E., Birol I., et al 2009, Circos: an information aesthetic for comparative genomics, Genome Res., 19, 1639–45. 19541911 10.1101/gr.092759.109 PMC2752132

[75] Han M.V. , Thomas G.W.C., Lugo-Martinez J., Hahn M.W. 2013, Estimating gene gain and loss rates in the presence of error in genome assembly and annotation using CAFE 3, Mol. Biol. Evol., 30, 1987–97. 23709260 10.1093/molbev/mst100

[76] Quevillon E. , Silventoinen V., Pillai S., Harte N. 2005, InterProScan: protein domains identifier, Nucleic Acids Res., 33, 116–20. 10.1093/nar/gki442 PMC1160203 15980438

[77] Vieira F.G. , Rozas J. 2011, Comparative genomics of the odorant-binding and chemosensory protein gene families across the Arthropoda: origin and evolutionary history of the chemosensory system, Genome Biol. Evol., 3, 476–90. 21527792 10.1093/gbe/evr033 PMC3134979

[78] Benton R. , Vannice K.S., Gomez-Diaz C., Leslie B.V. 2009, Variant ionotropic glutamate receptors as chemosensory receptors in *Drosophila*, Cell, 136, 149–62. 19135896 10.1016/j.cell.2008.12.001 PMC2709536

[79] Forêt S. , Wanner K.W., Maleszka R. 2007, Chemosensory proteins in the honey bee: insights from the annotated genome, comparative analyses and expressional profiling, Insect Biochem. Mol. Biol., 37, 19–28. 17175443 10.1016/j.ibmb.2006.09.009

[80] Dahanukar A. , Lei Y.T., Kwon J.Y., Carlson J.R. 2007, Two Gr genes underlie sugar reception in *Drosophila*, Neuron, 56, 503–16. 17988633 10.1016/j.neuron.2007.10.024 PMC2096712

[81] Freeman E.G. , Wisotsky Z., Dahanukar A. 2014, Detection of sweet tastants by a conserved group of insect gustatory receptors, Proc. Natl. Acad. Sci. U. S. A., 111, 1598–603. 24474785 10.1073/pnas.1311724111 PMC3910600

[82] Price M.N. , Dehal P.S., Arkin A.P. 2009, FastTree: computing large minimum-evolution trees with profiles instead of a distance matrix, Mol. Biol. Evol., 26, 1641–50. 19377059 10.1093/molbev/msp077 PMC2693737

[83] The Nasonia Genome Working Group. 2010, Functional and evolutionary insights from the genomes of three Nasonia species, Science, 327, 343–348. 20075255 10.1126/science.1178028 PMC2849982

[84] Graaf D.C. , Aerts M., Brunain M., et al 2010, Insights into the venom composition of the ectoparasitoid wasp *Nasonia vitripennis* from bioinformatic and proteomic studies, Insect Mol. Biol., 19, 11–26. 20167014 10.1111/j.1365-2583.2009.00914.x PMC3544295

[85] Gallie D.R. , Lewis N.J., Marzluff W.F. 1996, The histone 3′-terminal stem-loop is necessary for translation in Chinese hamster ovary cells, Nucleic Acids Res., 24, 1954–62. 8657580 10.1093/nar/24.10.1954 PMC145863

[86] Ye X.H. , Yan Z.C., Yang Y., et al 2020, A chromosome-level genome assembly of the parasitoid wasp *Pteromalus puparum*, Mol. Ecol. Resour., 20, 1384–402. 32562592 10.1111/1755-0998.13206

[87] Hughes A.L. 2014, Evolutionary diversification of insect innexins, J. Insect Sci., 14, 221. 25502029 10.1093/jisesa/ieu083 PMC5634128

[88] Huang Z. , Kawase-Koga Y., Zhang S., et al 2009, Transcription factor Lmo4 defines the shape of functional areas in developing cortices and regulates sensorimotor control, Dev. Biol., 327, 132–42. 19111533 10.1016/j.ydbio.2008.12.003 PMC2771174

[89] Sato S. , Omori Y., Katoh K., et al 2008, Pikachurin, a dystroglycan ligand, is essential for photoreceptor ribbon synapse formation, Nat. Neurosci., 11, 923–31. 18641643 10.1038/nn.2160

[90] Lazarević J. , Janković-Tomanić M. 2015, Dietary and phylogenetic correlates of digestive trypsin activity in insect pests, Entomol. Exp. Appl., 157, 123–51.

[91] Kim B.Y. , Lee K.S., Zou F.M., et al 2013, Antimicrobial activity of a honeybee (*Apis cerana*) venom Kazal-type serine protease inhibitor, Toxicon, 76, 110–7. 24076031 10.1016/j.toxicon.2013.09.017

[92] Negulescu H. , Guo Y., Garner T.P., et al 2015, A kazal-type serine protease inhibitor from the defense gland secretion of the subterranean termite *Coptotermes formosanus* Shiraki, PLoS One., 10, e0125376. 25978745 10.1371/journal.pone.0125376 PMC4433142

[93] Guo W. , Wu Z.X., Yang L.B., Cai Z.K., Zhao L.F., Zhou S.T. 2019, Juvenile hormone-dependent Kazal-type serine protease inhibitor Greglin safeguards insect vitellogenesis and egg production, FASEB J., 33, 917–27. 30063437 10.1096/fj.201801068R

[94] Zhao X.M. , Yang J.P., Gou X., Liu W.M., Zhang J.Z. 2021, Cuticular protein gene LmACP8 is involved in wing morphogenesis in the migratory locust, *Locusta migratoria*, J. Integr. Agr., 20, 1596–606.

[95] Zhou J.-J. , Vieira F.G., He X.-L., et al 2010, Genome annotation and comparative analyses of the odorant-binding proteins and chemosensory proteins in the pea aphid *Acyrthosiphon pisum*, Insect Mol. Biol., 19, 113–22. 10.1111/j.1365-2583.2009.00919.x 20482644

[96] Whiting A.R. 1967, The biology of the parasitic wasp *Mormoniella vitripennis* [=*Nasonia brevicornis*] (Walker), Q. Rev. Biol., 43, 333–406.

[97] Martinson E.O. , Mrinalini, Kelkar Y.D., Chang C.H., Werren J.H. 2017, The evolution of venom by co-option of single-copy genes, Curr. Biol., 27, 2007–13. 28648823 10.1016/j.cub.2017.05.032 PMC5719492

[98] Pikielny C.W. , Hasan G., Rouyer F., Rosbash M. 1994, Members of a family of *Drosophila* putative odorant-binding proteins are expressed in different subsets of olfactory hairs, Neuron, 12, 35–49. 7545907 10.1016/0896-6273(94)90150-3

[99] Gaudet P. , Livstone M., Lewis S.E., Thomas P. 2011, Phylogenetic-based propagation of functional annotations within the gene ontology consortium, Brief. Bioinform., 12, 449–62. 21873635 10.1093/bib/bbr042 PMC3178059

[100] Benton R. , Sachse S., Michnick S.W., Vosshall L.B. 2006, Atypical membrane topology and heteromeric function of *Drosophila* odorant receptors in vivo, PLoS Biol., 4, e20. 16402857 10.1371/journal.pbio.0040020 PMC1334387

[101] Sánchez-Gracia A. , Vieira F.G., Rozas J. 2009, Molecular evolution of the major chemosensory gene families in insects, Heredity (Edinb), 103, 208–16. 19436326 10.1038/hdy.2009.55

[102] Ai M. , Blais S., Park J.Y., Min S., Neubert T.A., Suh G.S. 2013, Ionotropic glutamate receptors IR64a and IR8a form a functional odorant receptor complex in vivo in *Drosophila*, J. Neurosci., 33, 10741–9. 23804096 10.1523/JNEUROSCI.5419-12.2013 PMC3693055

[103] Abuin L. , Bargeton B., Ulbrich M.H., Isacoff E.Y., Kellenberger S., Benton R. 2011, Functional architecture of olfactory ionotropic glutamate receptors, Neuron., 69, 44–60. 21220098 10.1016/j.neuron.2010.11.042 PMC3050028

[104] Yavuz A. , Jagge C., Slone J., Amrein H. 2014, A genetic tool kit for cellular and behavioral analyses of insect sugar receptors, Fly (Austin), 8, 189–96. 25984594 10.1080/19336934.2015.1050569 PMC4594417

[105] Jansen-González S. , Teixeira S.D.P., Pereira R.A.S. 2012, Mutualism from the inside: coordinated development of plant and insect in an active pollinating fig wasp, Arthropod Plant Interact., 6, 601–9.

[106] Jia L.Y. , Xiao J.H., Niu L.M., et al 2014, Delimitation and description of the immature stages of a pollinating fig wasp, Ceratosolen *s*olmsi marchali Mayr (Hymenoptera: Agaonidae). Bull. Entomol. Res., 104, 164–75. 24286501 10.1017/S0007485313000606

[107] Rana R. , Dahlman D., Webb B. 2002, Expression and characterization of a novel teratocyte protein of the braconid, *Microplitis croceipes* (Cresson), Insect Biochem. Mol. Biol., 32, 1507–16. 12530218 10.1016/s0965-1748(02)00071-1

[108] Diez-Roux G. , Ballabio A. 2005, Sulfatases and human disease, Annu. Rev. Genomics Hum. Genet., 6, 355–79. 16124866 10.1146/annurev.genom.6.080604.162334

[109] Wheelock C.E. , Shan G., Ottea J. 2005, Overview of carboxylesterases and their role in the metabolism of insecticides, J. Pestic. Sci., 30, 75–83.

[110] Yamamoto K. , Yamada N. 2016, Identification of a diazinon-metabolizing glutathione S-transferase in the silkworm, Sci. Rep., 6, 30073. 27440377 10.1038/srep30073 PMC4954967

[111] Pavlidi N. , Vontas J., Leeuwen T.V. 2018, The role of glutathione S-transferases (GSTs) in insecticide resistance in crop pests and disease vectors, Curr. Opin. Insect Sci., 27, 97–102. 30025642 10.1016/j.cois.2018.04.007

[112] Ishida Y. , Leal W.S. 2005, Rapid inactivation of a moth pheromone, Proc. Natl. Acad. Sci. USA., 102, 14075–9. 16172410 10.1073/pnas.0505340102 PMC1216831

[113] Scott J.G. 1999, Cytochrome P450 and insecticide resistance, Insect Biochem. Mol. Biol., 29, 757–77. 10510498 10.1016/s0965-1748(99)00038-7

[114] Schule R. M.A. , Berenbaum M.R. 2013, Structure and function of cytochrome P450s in insect adaptation to natural and synthetic toxins: insights gained from molecular modeling, J. Chem. Ecol., 39, 1232–45. 24036972 10.1007/s10886-013-0335-7

[115] The Honeybee Genome Sequencing Consortium. 2006, Insights into social insects from the genome of the honey bee *Apis mellifer*, Nature, 433, 931–49. 10.1038/nature05260 PMC2048586 17073008

[116] Bakalyar H.A. , Reed R.R. 1990, Identification of a specialized adenylyl cyclase that may mediate odorant detection, Science, 250, 1403–6. 2255909 10.1126/science.2255909

[117] Song C.H. , Hu C.D., Masago M., et al 2001, Regulation of a novel human phospholipase C, PLCε, through membrane targeting by Ras, J. Biol. Chem., 276, 2752–7. 11022048 10.1074/jbc.M008324200

[118] Suh P.G. , Park J.I., Manzoli L., et al 2008, Multiple roles of phosphoinositide-specific phospholipase C isozymes, BMB Rep., 41, 415–34. 18593525 10.5483/bmbrep.2008.41.6.415

[119] Leinders-Zufall T. , Chamero P. 2016, Cyclic GMP signaling in olfactory sensory neurons, 141–155, in: Zuefall F. and Munger S.D., eds. Chemosensory Transduction, The Dectection of Odors, Tastes, and Other Chemostimuli. London, UK: Academic Press of Elsevier.

[120] Ferguson C.H. , Zhao H.Q., Cyclic A.M.P. 2016, Signaling in the main olfactory epithelium, 123–140, in Zuefall F., Munger S.D., eds. Chemosensory Transduction. The Detection of Odors, Tastes, and Other Chemostimuli. London, UK: Academic Press Elsevier.

[121] Firestein S. , Shepherd G.M., Werblin F. 1990, Time course of the membrane current underlying sensory transduction in salamander olfactory receptor neurons, J. Physiol., 430, 135–58. 2086763 10.1113/jphysiol.1990.sp018286 PMC1181732

[122] Zufall F. , Hatt H. 1991, Dual activation of a sex pheromone-dependent ion channel from insect olfactory dendrites by protein kinase C activators and cyclic GMP, Proc. Natl. Acad. Sci. USA., 88, 8520–4. 1717980 10.1073/pnas.88.19.8520 PMC52540

[123] Boekhoff I. , Michel, W.C., Breer, H., Hatt H. 1994, Single odors differentially stimulate dual second messenger pathways in lobster olfactory receptor cells, J. Neurosci., 14, 3304–9. 8182473 10.1523/JNEUROSCI.14-05-03304.1994 PMC6577444

[124] Ache B.W. , Zhainazarov A. 1995, Dual second-messenger pathways in olfactory transduction, Curr. Opin. Neurobiol., 5, 461–6. 7488847 10.1016/0959-4388(95)80006-9

