# Supplementary material for: A chromosome-level genome assembly of the pollinating fig wasp Valisia javana
Source: DNA Res. 2022 May 20;29(3):dsac014. doi: 10.1093/dnares/dsac014 (PMC9160881; doi:10.1093/dnares/dsac014)
Supplement: dsac014_Supplementary_Data [file dsac014_supplementary_data.zip › Readme Chen L. F. et al..docx]

A chromosome-level genome assembly of the pollinating fig wasp *Valisia javana*

**Running title**: Genome assembly of a fig wasp

Lianfu Chen^#1,2^, Chao Feng^#1^, Rong Wang^3^, Nong Xiaojue^1^, Deng xiaoxia^1^, Xiaoyong Chen^3^*, Hui Yu^1,2^*

**^1^** Key Laboratory of Plant Resource Conservation and Sustainable Utilization, South China Botanical Garden, The Chinese Academy of Sciences, Guangzhou 510650, China;

**^2^** Guangdong Provincial Key Laboratory of Applied Botany, South China Botanical Garden, The Chinese Academy of Sciences, Guangzhou 510650, China;

**^3^** Southern Marine Science and Engineering Guangdong Laboratory (Guangzhou), Guangzhou 511458;

**^4^** School of Ecological and Environmental Sciences, Tiantong National Station for Forest Ecosystem Research, East China Normal University, Shanghai 200241, China;

**^#^** These authors contributed equally: Lianfu Chen, Chao Feng. ***** Correspondence: [xychen@des.ecnu.edu.cn](mailto:xychen@des.ecnu.edu.cn) (X. Y. C.), [yuhui@scbg.ac.cn](mailto:yuhui@scbg.ac.cn) (H. Y.), Tel.: +886-021-54345469 (X. Y. C.); Tel.: +886-020-37252759 (H. Y.)

# Supplemental information

Additional supporting information may be found online in the Supporting Information section.

Table S1. Species origin and genome assembly of 30 insect species.

Table S2. Genome sequencing of *V. javana* using Illumina and PacBio.

Figure S1. Estimate of the *V. javana g*enome size with 21-mer.

**Table S3. Statistics of** *V. javana* **genome assemblies.**

Figure S2. BUSCO assessments for 30 insect species. For ***V. javana*, 5,516 out of 5,991 (92.1%) conserved Hymenoptera genes were found in the whole genome of it.**

**Table S4.** Information of telomere in the 13 contigs of *V. javana* genome.

Table S5. Repeat classes identified in the *V. javana* genome.

Table S6. Comparison of the proportion of repetitive sequences in genome among 30 insect species.

Table S7. Annotation of non-coding RNA in the *V. javana* genome.

Table S8. Comparison of genome annotation among 30 insect species.

Table S9. Functional annotation of *V. javana* genome.

Table S10. Detailed information about the PSG genes in genome of *V. javana* and Agaodinae (including four fig wasp species).

Table S11. Detailed information about the expansion and contraction of gene family in genome of *V. javana* and Agaonidae (including four fig wasp species).

Figure S3. Gene tree constructed from all odorant binding protein (OBPs) of four fig wasp species and *Drosophila melanogaster* (orange). The diamond pattern indicated that the genes were highly expressed in transcriptome data of *V. javana*.

Table S12. Statistics of RNA-Seq data of *V. javana*

Table S13. The number of chemosensory genes, odorant binding protein (OBP), chemosensory protein (CSP), olfactory receptor (OR), ionic receptor (IR), and gustatory receptor (GR), and venom genes for 30 insect species.

Table S14. The expression of chemosensory genes, odorant binding proteins (OBPs), chemosensory proteins (CSPs), odorant receptors (ORs), ionic receptors (IRs) and gustatory receptors (GRs) in five samples of transcriptome of *V. javana*. A, B, C represent the controls; E and F represent two samples stimulated by odors.

Figure S4. Gene tree constructed from all chemosensory proteins (CSPs) of four fig wasp species, *Apis mellifera* (cyan) and *Drosophila melanogaster* (orange). CSPs in four fig wasp species can be divided into 8 groups contained genes from all four fig wasps. The diamond pattern indicated that the genes were highly expressed in transcriptome data of *V. javana*.

Figure S5. Gene tree constructed from all odorant receptors (ORs) of four fig wasp species and *Drosophila melanogaster* (orange). The diamond pattern indicated that the genes were highly expressed in transcriptome data of *V. javana*.

Figure S6. Gene tree constructed from all ionotropic receptors (IRs) of four fig wasp species, *Apis mellifera* (cyan) and *Drosophila melanogaster* (orange). The diamond pattern indicated that the genes were highly expressed in transcriptome data of *V. javana*.

Figure S7. Gene tree constructed from all gustatory receptors (GRs) of four fig wasp species, *Nasonia vitripennis* (black), *Apis mellifera* (cyan) and *Drosophila melanogaster* (orange). The diamond pattern indicated that the genes were highly expressed in transcriptome data of *V. javana*.

Table S15. Comparison of the number of detoxification genes and venom genes among 30 insect species.

Table S16. Comparison of the number of detail entries of venom genes among 30 insect species.

Figure S8. Gene tree constructed from all cytochrome P450s (P450) genes of four fig wasp species, *Nasonia vitripennis* and *Apis mellifera.* Different clades are marked as different colors.

Figure S9. Gene tree constructed from all Glutathione-S-transferases (GST) genes of four fig wasp species, *Nasonia vitripennis* and *Apis mellifera.* Different clades are marked as different colors.

Figure S10. Gene tree constructed from all carboxylesterases (CCE) genes of four fig wasp species, *Nasonia vitripennis* and *Apis mellifera.* Different clades are marked as different colors.

Figure S11. An overview of olfactory transduction pathway (ko04740) unigenes and differentially expressed genes in *V. javana* transcriptome between samples stimulated by host odors and the controls. Grey indicates the unigenes annotated in *V. javana*; green represents a significantly down regulated unigenes in samples stimulated by host odors.

Figure S12. An overview of cAMP signaling pathway (ko04024) unigenes and differentially expressed genes in *V. javana* transcriptome between samples stimulated by host odors and the controls. Grey indicates the unigenes annotated in *V. javana*; green represents a significantly down regulated unigenes in samples stimulated by host odors.

Figure S13. An overview of cGMP-PKG signaling pathway (ko04022) unigenes and differentially expressed genes in *V. javana* transcriptome between samples stimulated by host odors and the controls. Grey indicates the unigenes annotated in *V. javana*; green represents a significantly down regulated unigenes in samples stimulated by host odors.

Figure S14. An overview of calcium signaling pathway (ko04020) unigenes and differentially expressed genes in *V. javana* transcriptome between samples stimulated by host odors and the controls. Grey indicates the unigenes annotated in *V. javana*; green represents a significantly down regulated unigenes in samples stimulated by host odors.

Figure S15. An overview of Ras signaling pathway (ko04014) unigenes and differentially expressed genes in *V. javana* transcriptome between samples stimulated by host odors and the controls. Grey indicates the unigenes annotated in *V. javana*; green represents a significantly down regulated unigenes in samples stimulated by host odors.

Figure S16. An overview of Rap1 signaling pathway (ko04015) unigenes and differentially expressed genes in *V. javana* transcriptome between samples stimulated by host odors and the controls. Grey indicates the unigenes annotated in *V. javana*; green represents a significantly down regulated unigenes in samples stimulated by host odors.
