# Supplementary figures and images for: A chromosome-level genome assembly of the pollinating fig wasp Valisia javana
Source: DNA Res. 2022 May 20;29(3):dsac014. doi: 10.1093/dnares/dsac014 (PMC9160881; doi:10.1093/dnares/dsac014)

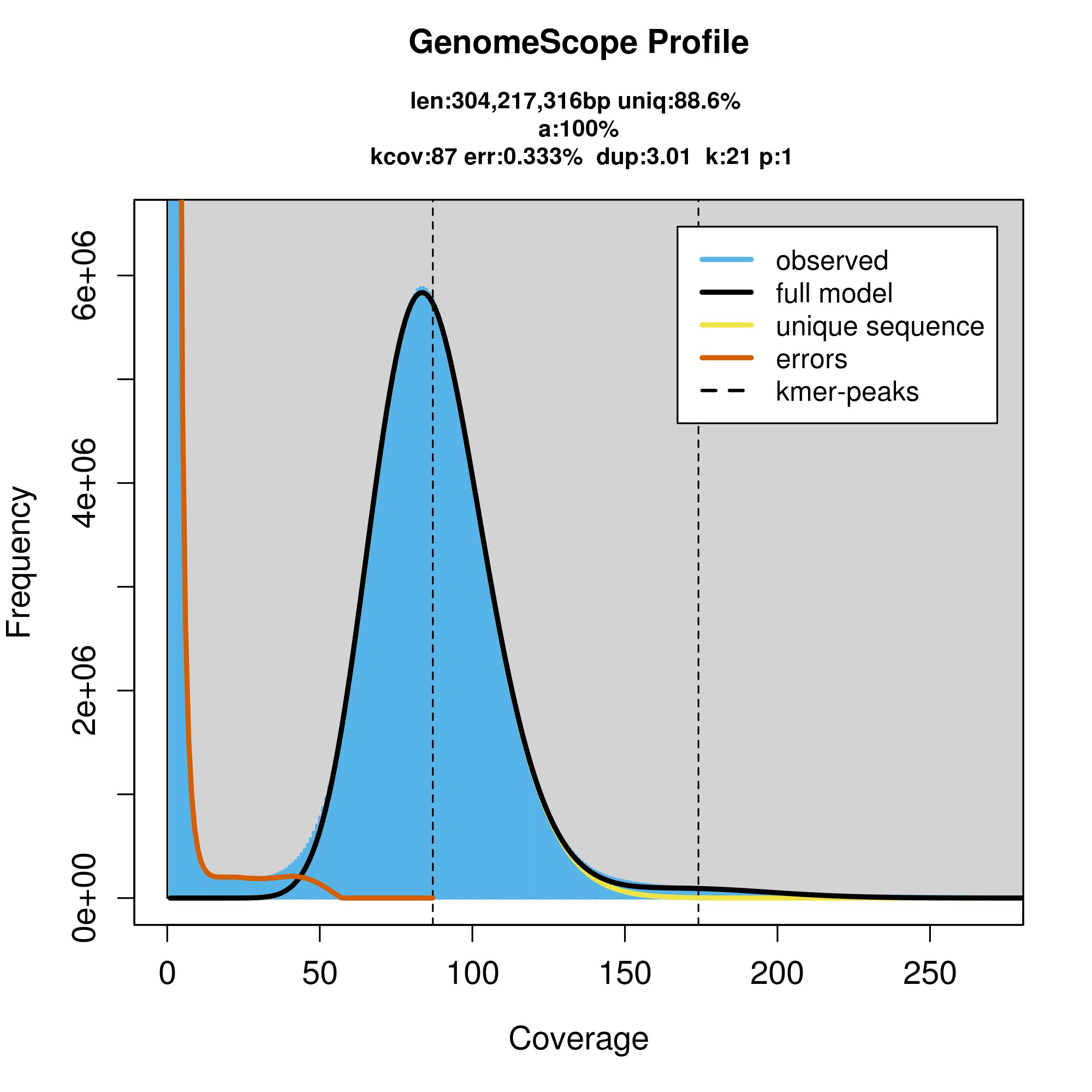

Supplement: dsac014_Supplementary_Data [file dsac014_supplementary_data.zip › Figure S1. Estimate of the V. javana genome size with 21-mer.jpg]

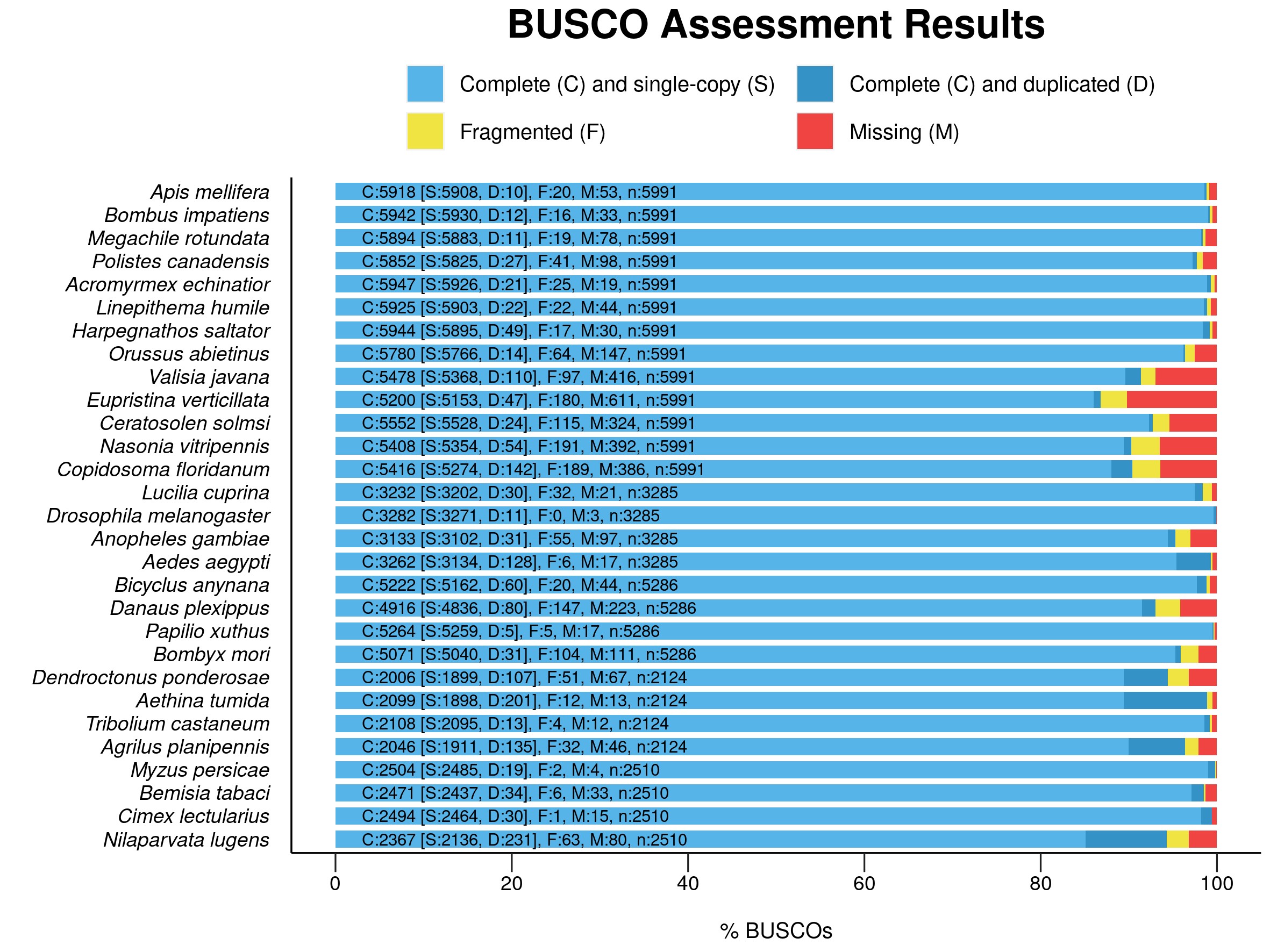

Supplement: dsac014_Supplementary_Data [file dsac014_supplementary_data.zip › Figure S2. BUSCO assessments for 30 insect species.jpg]

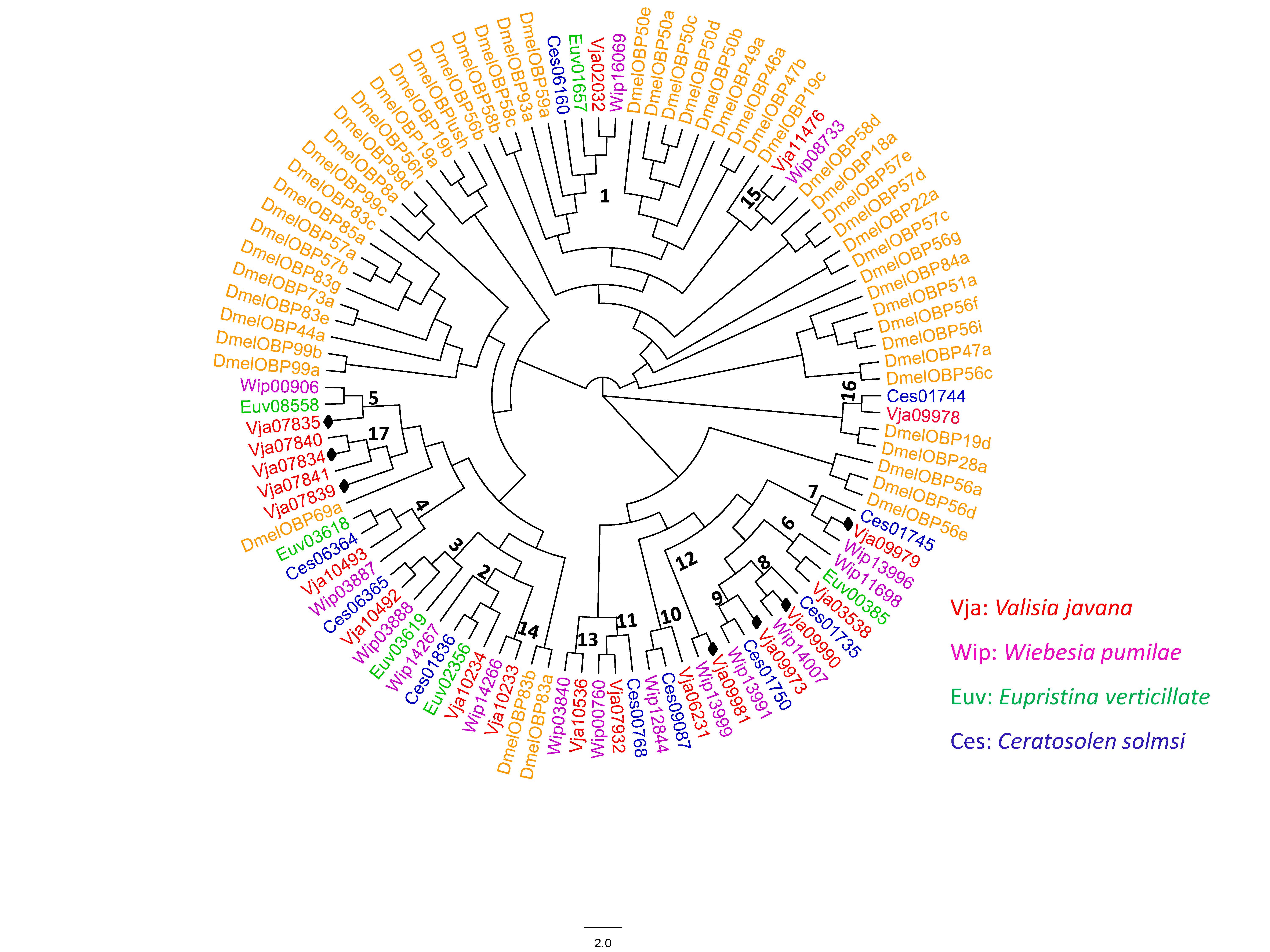

Supplement: dsac014_Supplementary_Data [file dsac014_supplementary_data.zip › Figure S3. Gene tree constructed from OBPs.jpg]

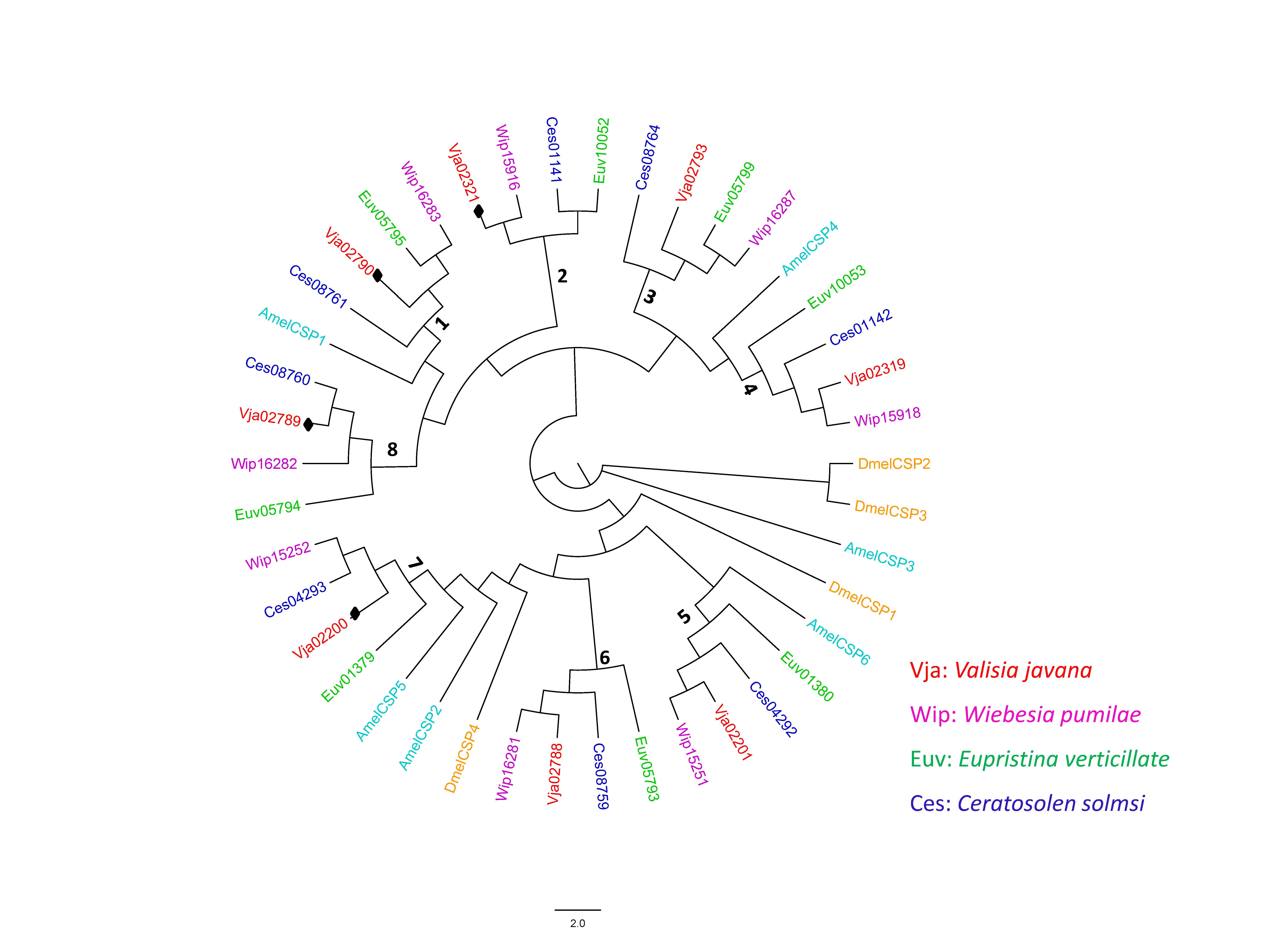

Supplement: dsac014_Supplementary_Data [file dsac014_supplementary_data.zip › Figure S4. Gene tree constructed from CSPs.jpg]

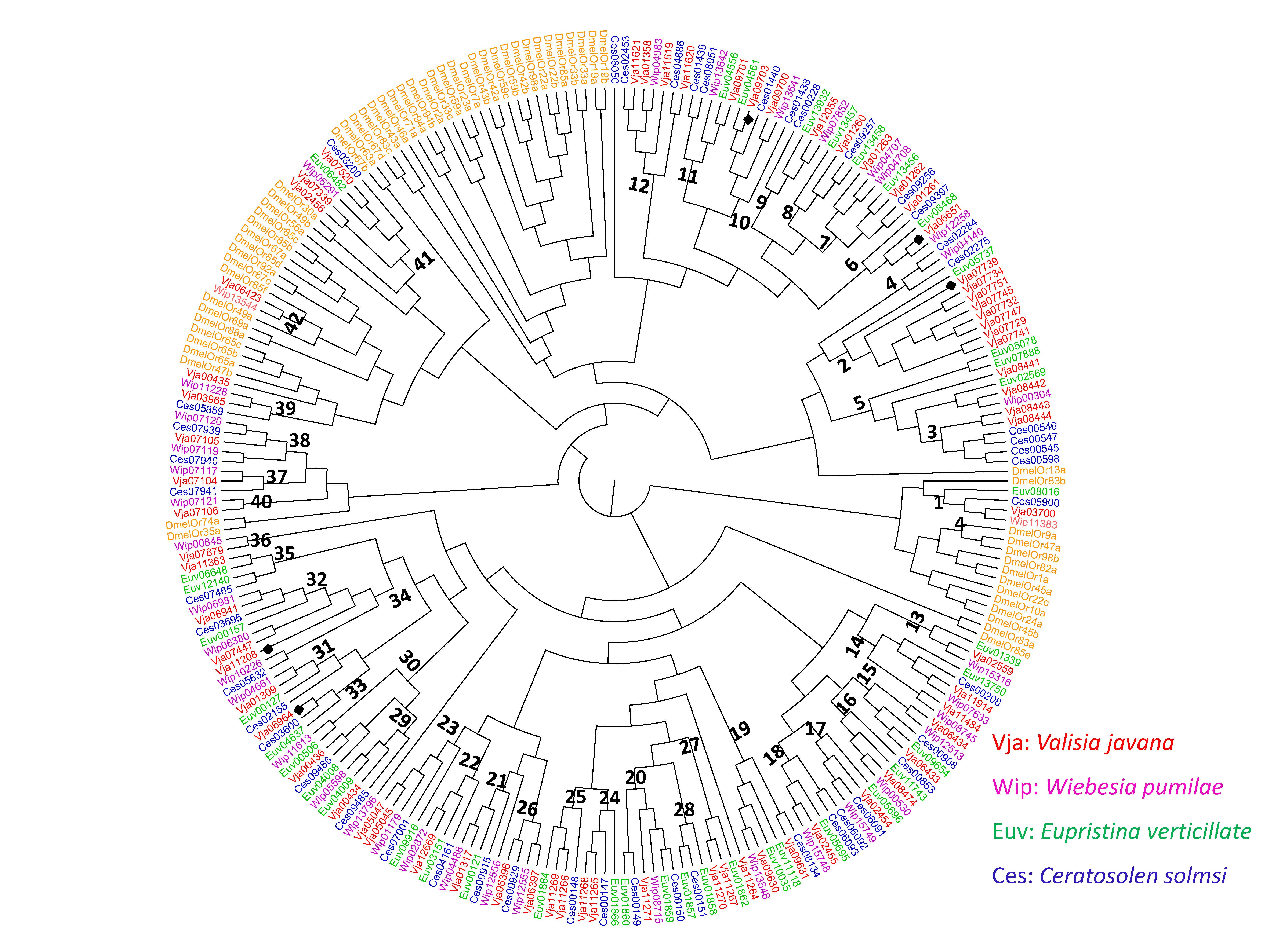

Supplement: dsac014_Supplementary_Data [file dsac014_supplementary_data.zip › Figure S5. Gene tree constructed from ORs.jpg]

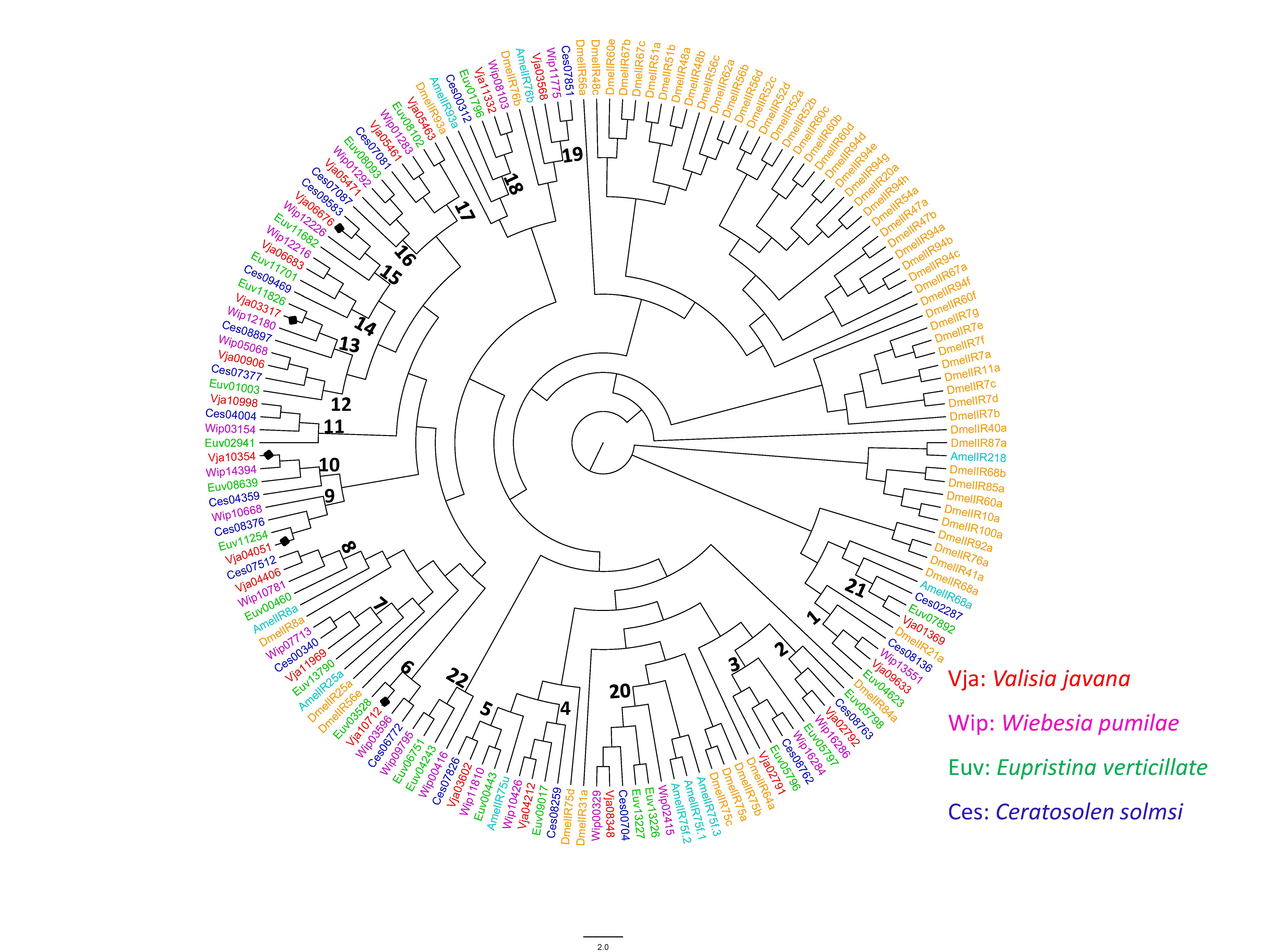

Supplement: dsac014_Supplementary_Data [file dsac014_supplementary_data.zip › Figure S6. Gene tree constructed from IRs.jpg]

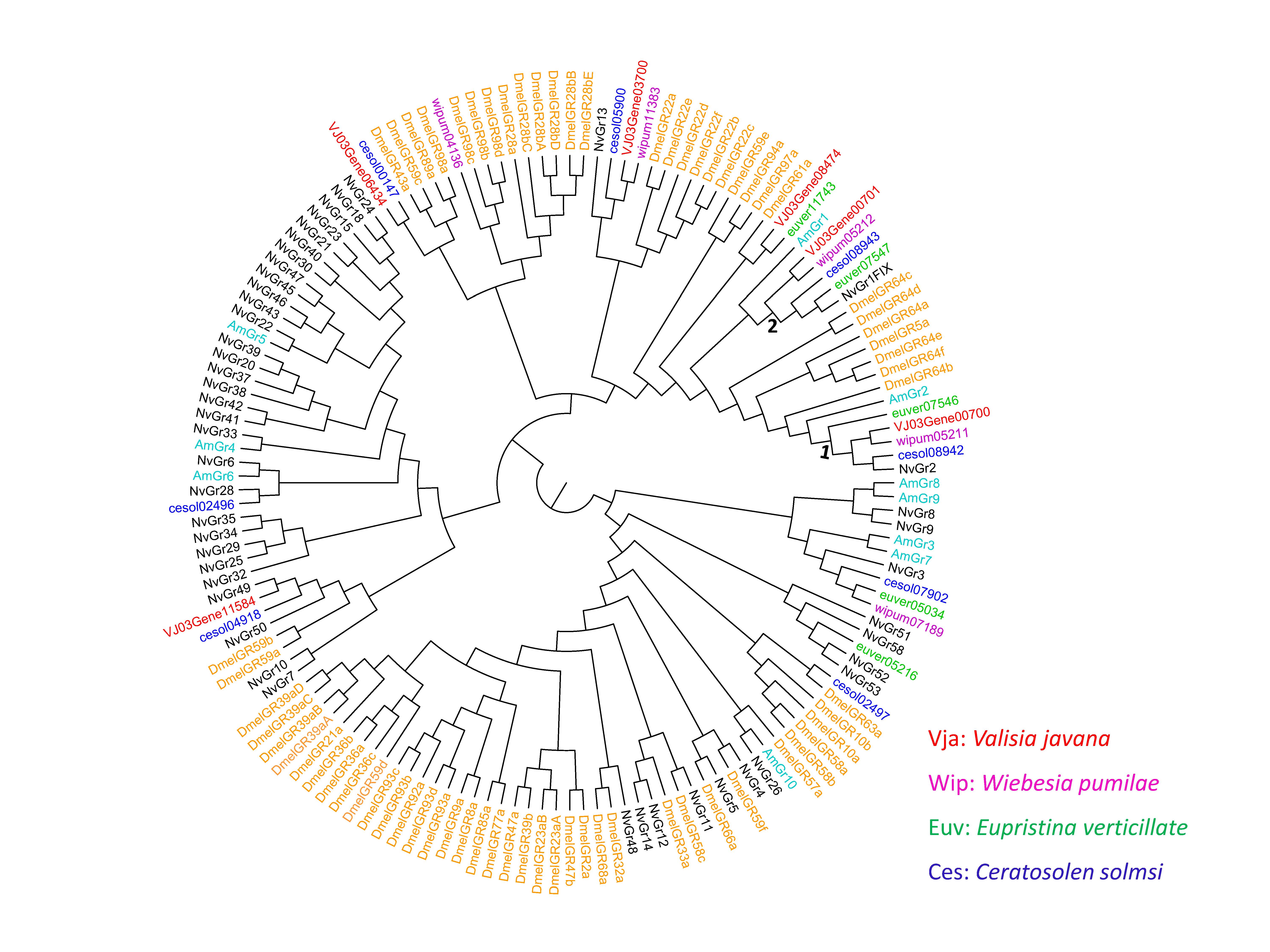

Supplement: dsac014_Supplementary_Data [file dsac014_supplementary_data.zip › Figure S7.Gene tree constructed from GRs.jpg]

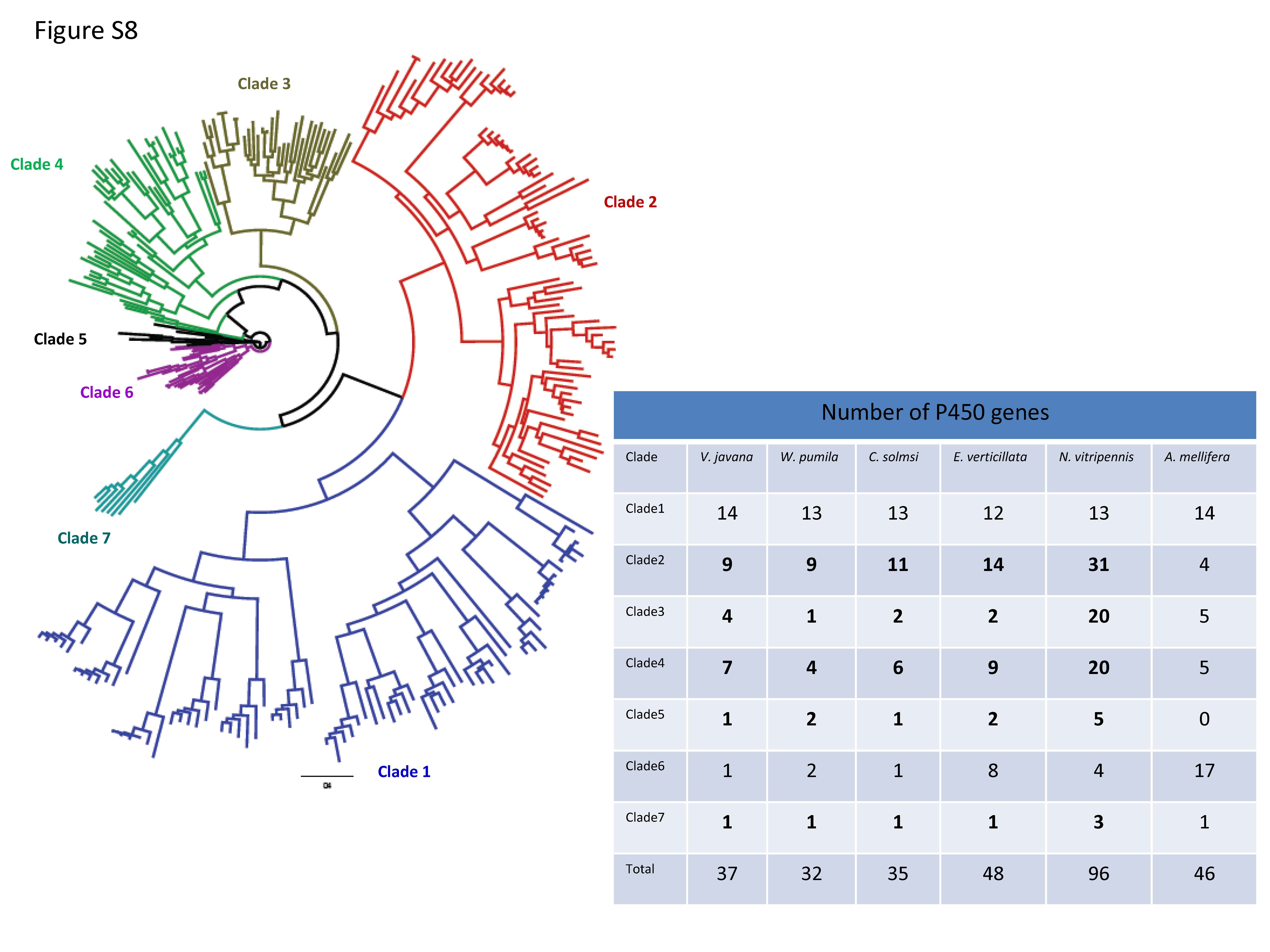

Supplement: dsac014_Supplementary_Data [file dsac014_supplementary_data.zip › Figure S8-P450 tree.jpg]

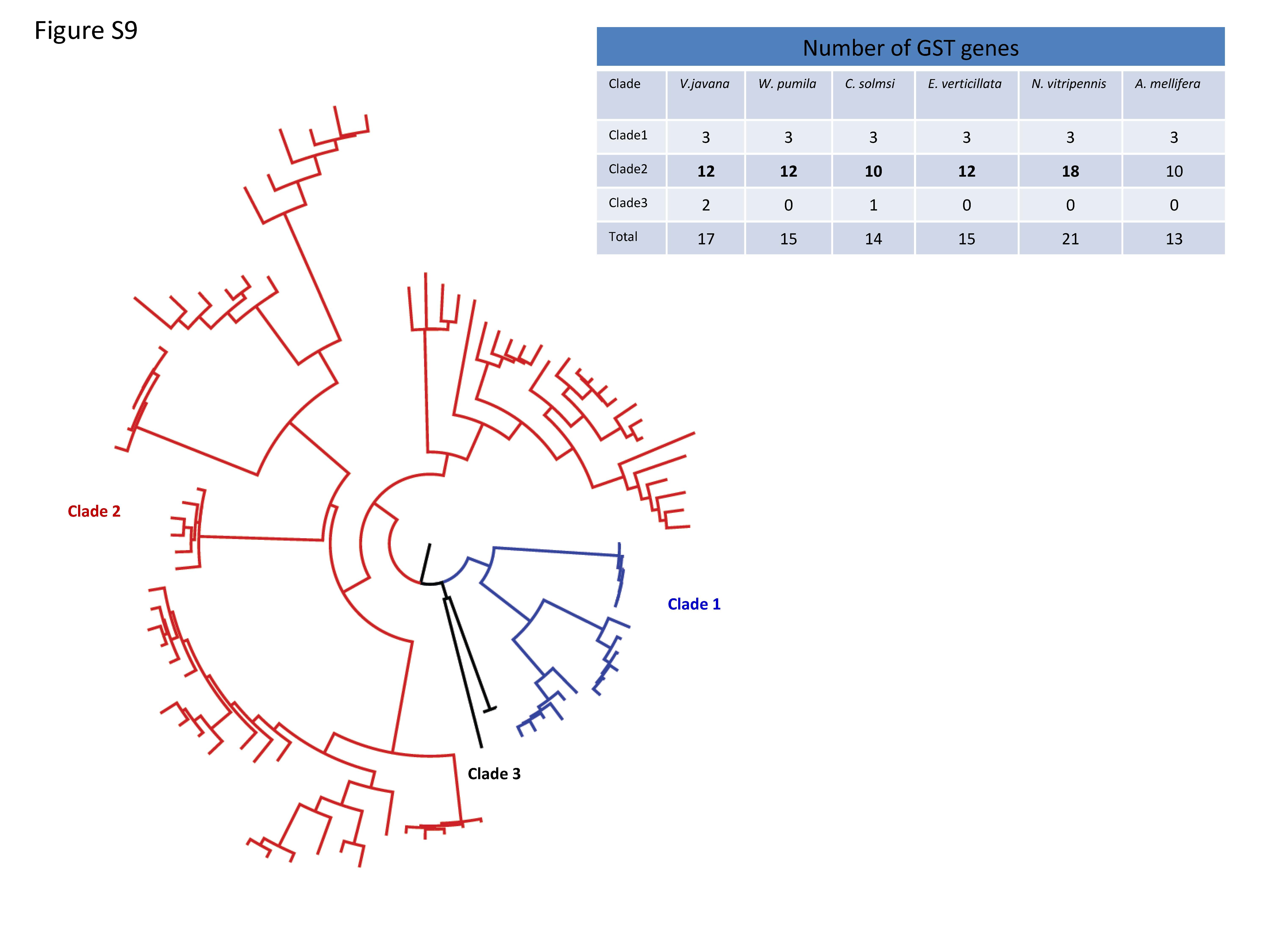

Supplement: dsac014_Supplementary_Data [file dsac014_supplementary_data.zip › Figure S9-GST tree.jpg]

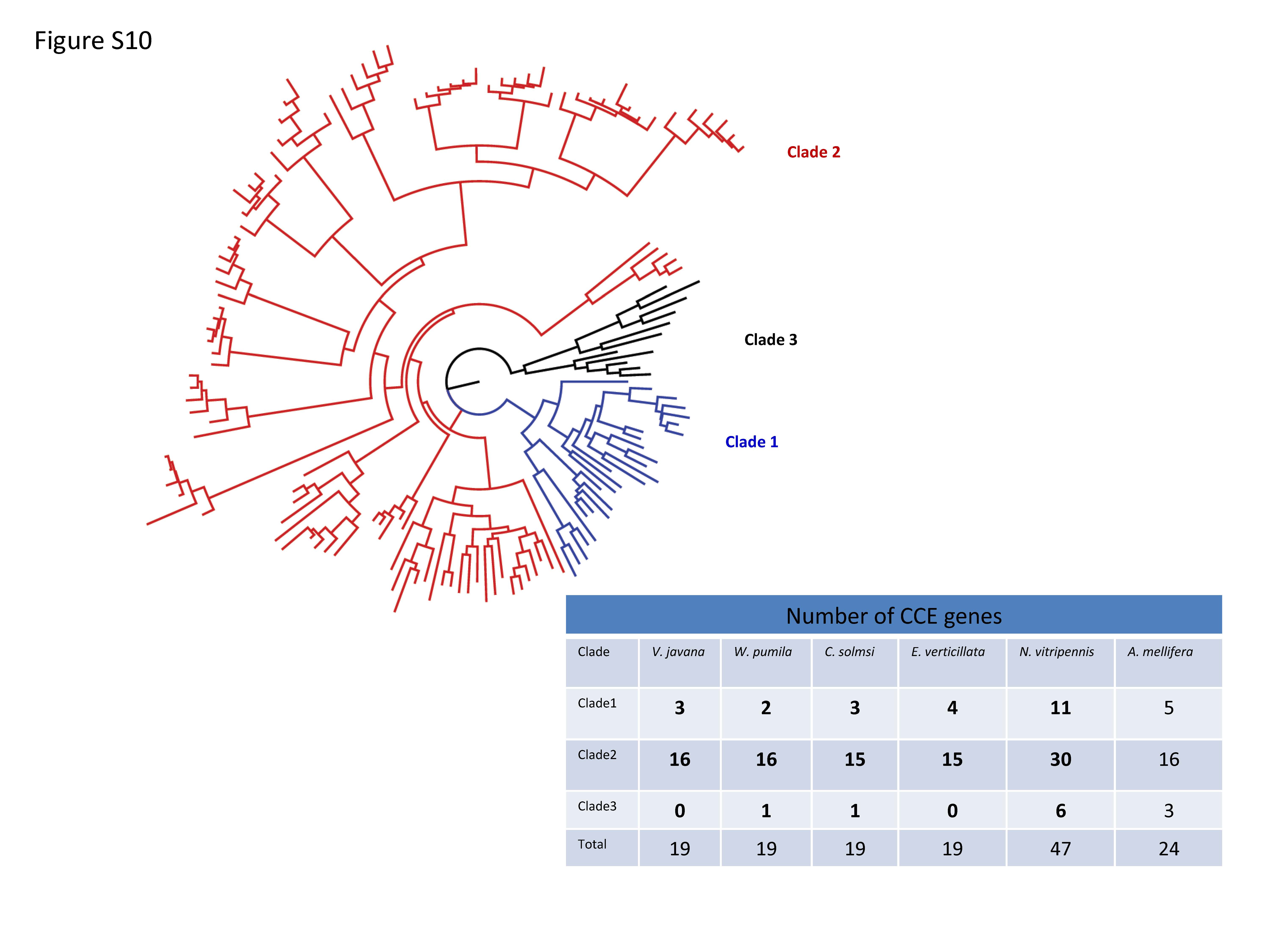

Supplement: dsac014_Supplementary_Data [file dsac014_supplementary_data.zip › Figure S10-CCE tree.jpg]

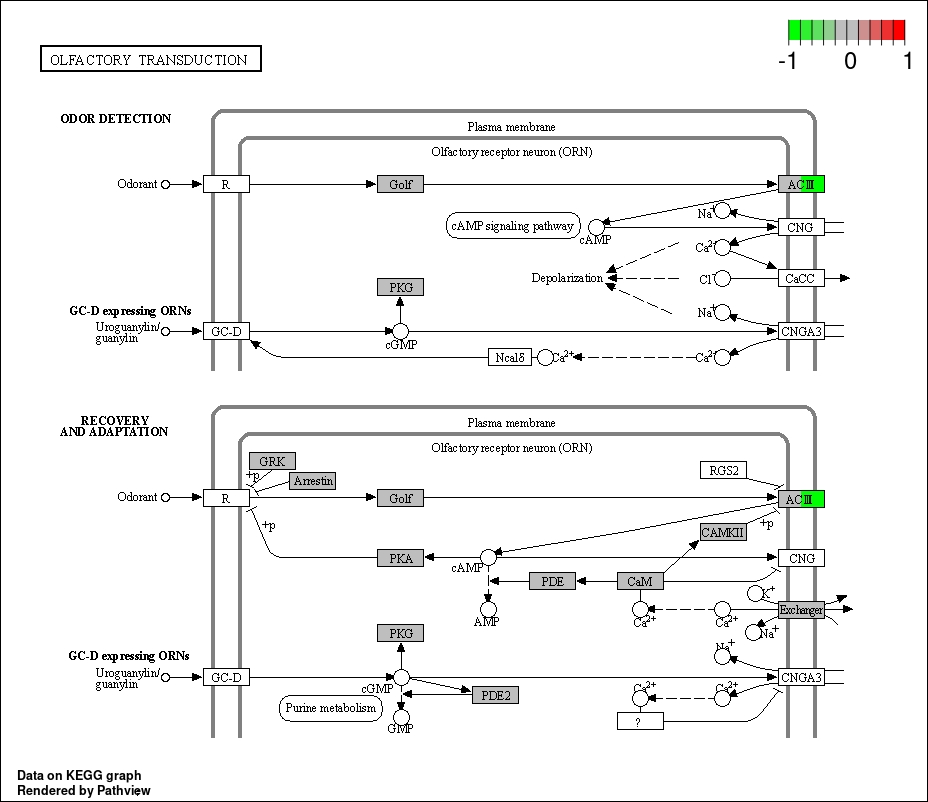

Supplement: dsac014_Supplementary_Data [file dsac014_supplementary_data.zip › Figure-S11. An overview of olfactory transduction ko04740.jpg]

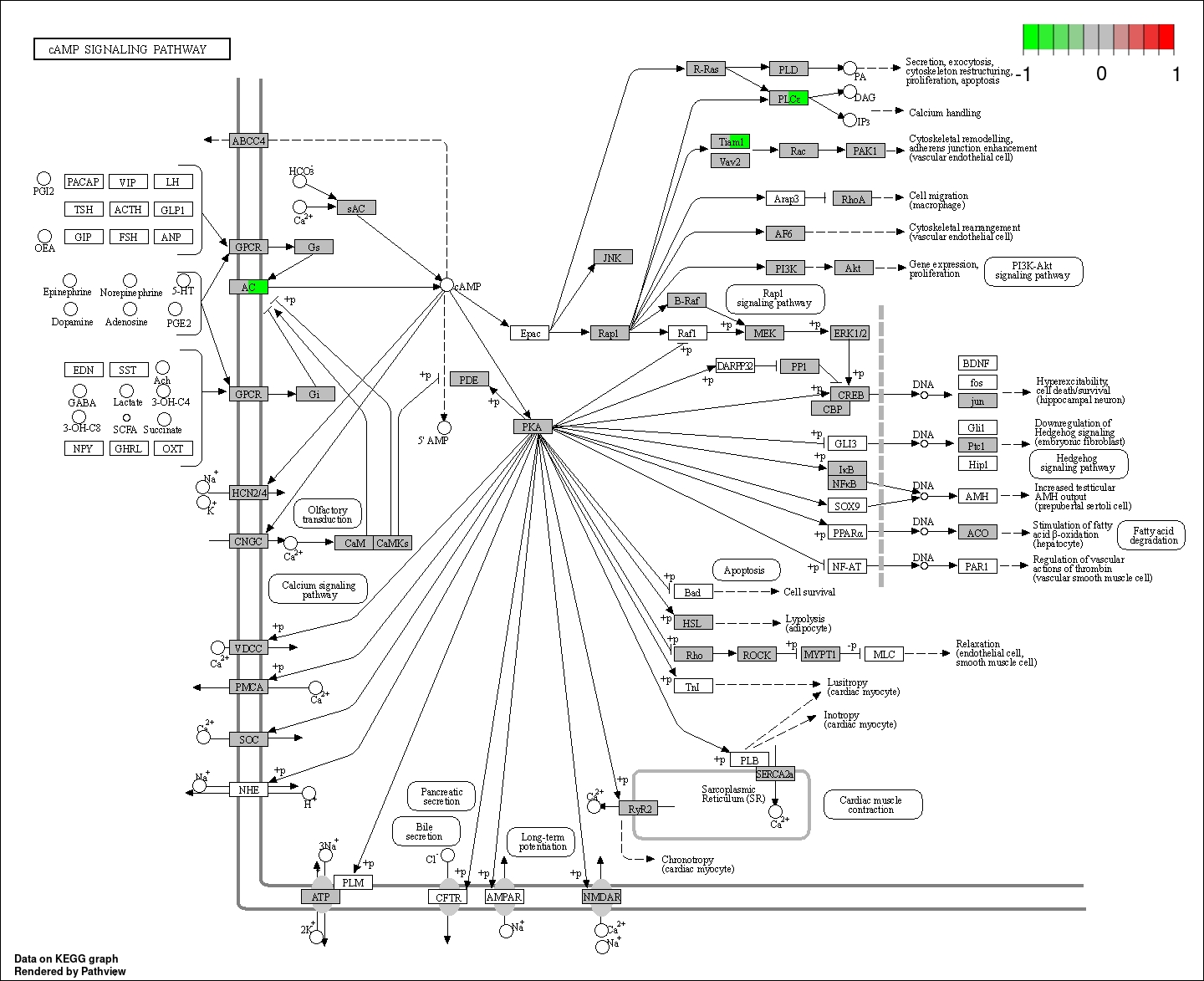

Supplement: dsac014_Supplementary_Data [file dsac014_supplementary_data.zip › Figure-S12. An overview of cAMP signaling pathway ko04024.jpg]

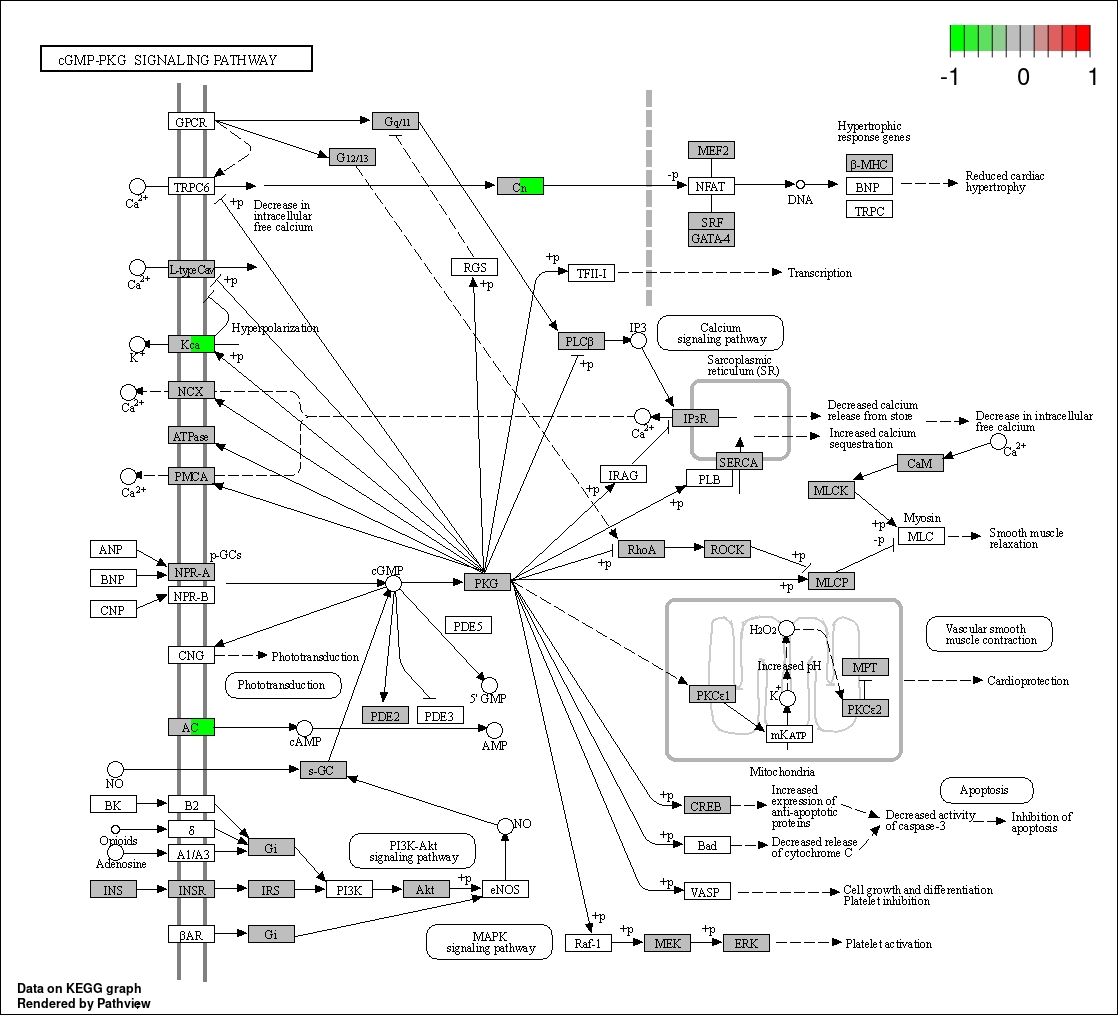

Supplement: dsac014_Supplementary_Data [file dsac014_supplementary_data.zip › Figure-S13. An overview of cGMP-PKG signaling pathway ko04022.jpg]

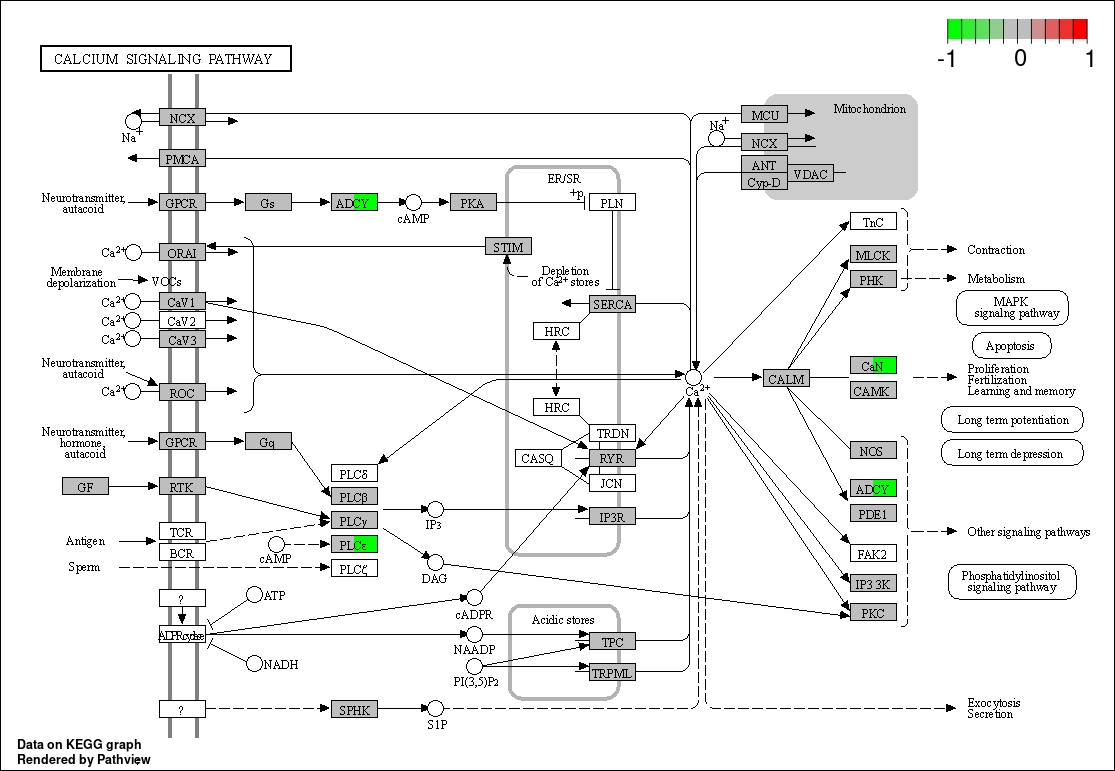

Supplement: dsac014_Supplementary_Data [file dsac014_supplementary_data.zip › Figure-S14. An overview of calcium signaling pathway ko04020.jpg]

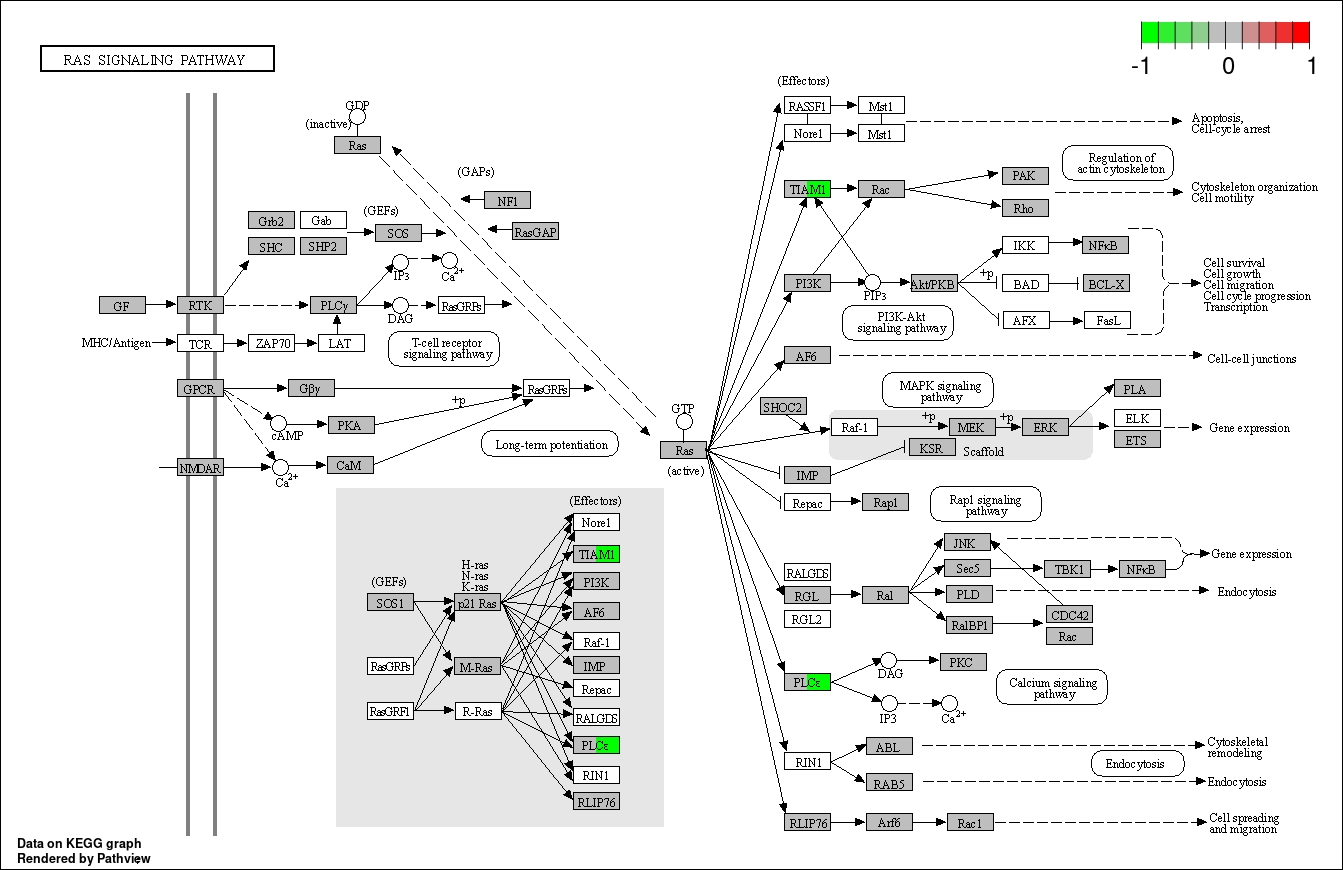

Supplement: dsac014_Supplementary_Data [file dsac014_supplementary_data.zip › Figure-S15. An overview of Ras signaling pathway ko04014.jpg]

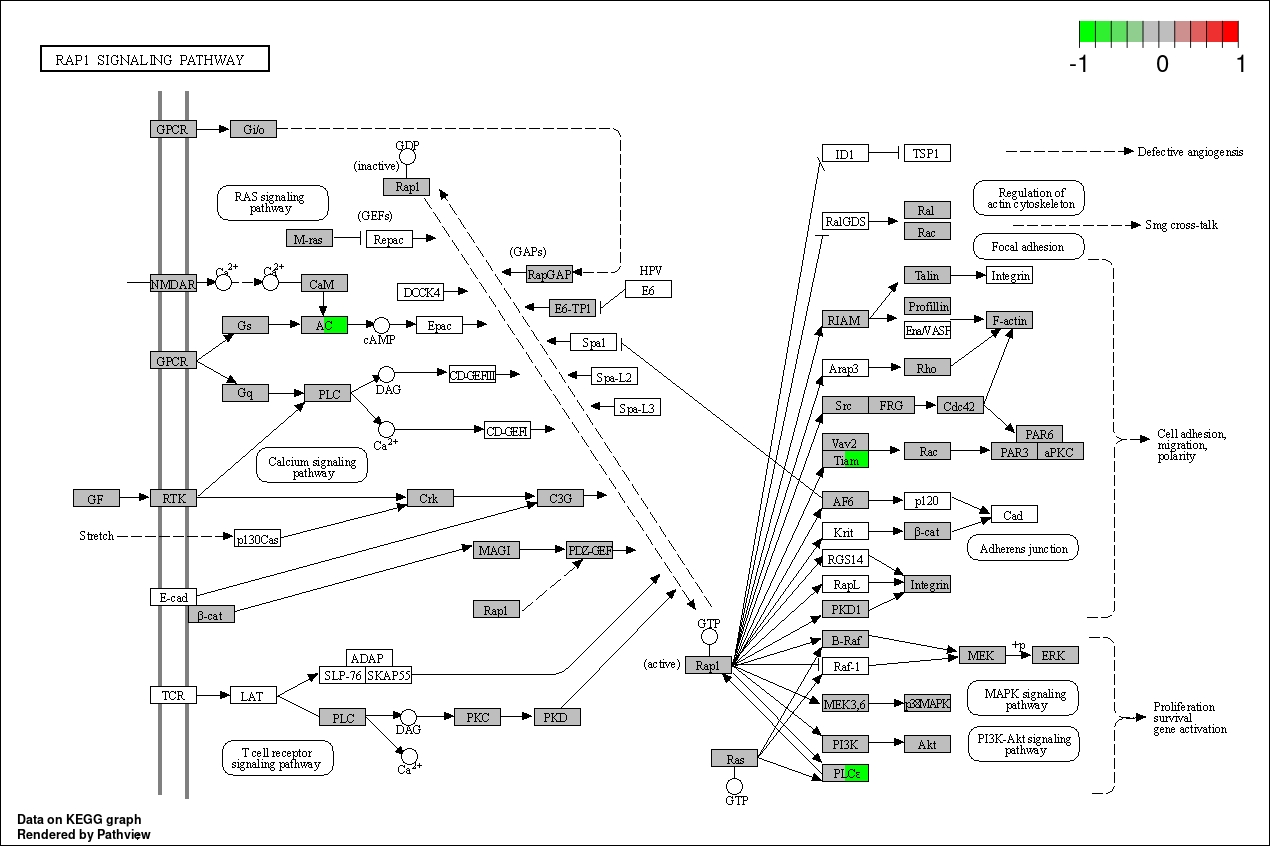

Supplement: dsac014_Supplementary_Data [file dsac014_supplementary_data.zip › Figure-S16. An overview of Rap1 signaling pathway ko04015.jpg]
